# The effects of environmental prenatal program on environmental health perception and behavior using internet-based intervention in South Korea: A non-randomized controlled study

**DOI:** 10.1371/journal.pone.0277501

**Published:** 2022-11-15

**Authors:** Hyun Kyoung Kim, Geum Hee Jeong, Hye Young Min

**Affiliations:** 1 Department of Nursing, Kongju National University, Gongju, South Korea; 2 School of Nursing and Research Institute in Nursing Science, Hallym University, Chuncheon, South Korea; 3 College of Nursing, Ewha Womans University, Seoul, South Korea; Public Library of Science, UNITED KINGDOM

## Abstract

**Purpose:**

This study aimed to develop and examine the effects of an internet-based intervention program on environmental perception and behavior among Korean pregnant women based on revised protection motivation theory.

**Method:**

This study was a non-equivalent control group pre-post-test design. The experimental program consisted of prenatal education, reduction of fine dust, birth education, environmental health promotion, and postnatal management education using zoom video conferences. The face-to-face interventions were provided through regular prenatal classes at public health services for the control group. The total participant was 49 pregnant women: 25 in the experimental group and 24 in the control group. The program adaptation was conducted between April 2021 and November 2021 in Korea. The data were analyzed by ANCOVA and t-test to examine the effects using SPSS 26.0 program.

**Results:**

After intervention of the program, environmental severity (F = 17.96, p < .001), response efficacy (F = 15.69, p < .001), and total environmental perception (F = 7.80, p = .008) were higher in the experimental group than in the control group. There were no significant differences in feasibility, accessibility, satisfaction, susceptibility, self-efficacy, barrier, personal environmental behavior, and community environmental behavior between the two groups.

**Conclusion:**

The internet-based educational program can be the alternative for the face-to-face prenatal class to promote environmental health perceptions during pregnancy in the pandemic situations.

## Introduction

Climate change and environmental pollution exacerbate the effects of environmental toxicants on human health. Environmental toxins refer to chemicals, heavy metals, electromagnetic waves, and radiation that harm the human body via water, food, air, and soil [1]. In particular, women are sensitive to environmental toxins due to their higher number of hormone receptors with which environmental toxins are capable of interacting. Women are especially vulnerable to environmental toxins during pregnancy, and environmental toxins absorbed through the placenta can cause fetal problems [2]. Pregnancy is a critical window in a woman’s life cycle regarding exposure to environmental toxins [1].

The effects of environmental toxins on pregnant women and fetuses have been studied in recent years, including the effects of endocrine disruptors (EDCs) on pregnant women and fetuses. The rate of preterm births was 1.6 times higher among women who consumed drinking water contaminated with parabens and bisphenol A [3]. The consumption of lead-contaminated water lowered the overall fertility rate and increased the number of low-birthweight infants [4]. Exposure to electromagnetic waves during early pregnancy was associated with an increase in spontaneous abortion [5]. In fetuses, EDCs caused issues with psychomotor development and reproductive health [6]. Bisphenol A, phthalate, and pesticide exposure decreased the number of primordial follicles in fetuses [7]. As fetuses grow, phthalates shorten the fetal femur and reduce the weight of newborns and children aged 2 to 5 years [8]. Exposure to pesticides during pregnancy slowed children’s cognitive development [9].

Healthcare providers have a responsibility to provide education to prevent environmental health problems in pregnant women and fetuses [2]. Traditionally, South Korea has a prenatal culture in which prenatal education (*taegyo*) is provided to protect the mother and fetus from environmental toxins [10]. Currently, antenatal education is conducted at public health centers, hospitals, and private institutions. Existing prenatal education programs focus on understanding physiological changes, prenatal care, childbirth, postpartum care, and newborn care. Few prenatal education programs include relevant information to protect mothers and fetuses from environmental toxins [11]. As a study on environmental health behavior interventions for pregnant women reported, environmental interventions that used cartoons [12] and kiosks [13] were accompanied by increases in environmental knowledge, perception, and behavior.

Due to the coronavirus disease 2019 (COVID-19) pandemic, which has continued for more than 2 years, social distancing has become mandatory, and few face-to-face education programs have been conducted [14]. Internet-based education interventions, a representative method of non-face-to-face education, refer to interventions in which educators and learners develop and share educational content via internet [15]. There are various methods of conducting internet-based educational interventions for pregnant women, including web-based interventions, which constitute a method for learners to access educational programs through websites [16]. In recent years, the use of mobile phone applications for health purposes has grown [17].

Over the past 10 years, internet-based prenatal education has been found to affect depression, sleep, nutrition, efficacy, and breastfeeding [18]. Pregnant women are sensitive to environmental pollution and need environmental health education to protect themselves from environmental risk factors [11]. Therefore, this study developed and investigated the effects of an internet-based intervention to promote positive environmental health behaviors in pregnant women. The purpose of this study was to confirm that an environmental prenatal education program using an internet-based intervention affected pregnant women’s environmental health perceptions and behaviors. The specific research objectives were as follows: first, to develop an internet-based environmental prenatal program (IEPP) for pregnant women; second, to investigate the effect of an IEPP on environmental health perceptions and behaviors; and third, to investigate the effects of an IEPP on educational satisfaction. The hypotheses were as follows:

Hypothesis 1: The control and experimental groups will have different scores for environmental health perceptions after an IEPP.
Hypothesis 2: The control and experimental groups will have different scores for environmental health behaviors after an IEPP.
Hypothesis 3: The control and experimental groups will have different scores for educational satisfaction after an IEPP.

## Materials and methods

### 1. IEPP development

This study used a methodological framework that followed the five stages of the Analysis, Design, Development, Implementation, and Evaluation (ADDIE) model [19]. First, during the analysis stage, the content of the IEPP was structured using a literature review of studies on environmental health interventions. An advanced search of abstracts and titles was conducted from April 2 to 13, 2021, for the terms: ’(((("Internet-Based Intervention"[Mesh]) OR "Education, Distance"[Mesh]) OR "Mobile Applications"[Mesh]) AND "Environment*"[Mesh]) AND "Birth*"[Mesh] OR "Preg*"[Mesh] OR "Prenat*"[Mesh] OR "Antenat*"[Mesh],’ (’internet-based intervention’ OR ’education, distance’ OR ’mobile applications’) AND ’environment*’ AND (’birth*’ OR ’preg*’ OR ’prenat*’ OR ’antenat*’), (’internet-based intervention’ OR ’education, distance’ OR ’mobile applications’) AND ’environment*’ AND (’birth*’ OR ’preg*’ OR ’prenat*’ OR ’antenat*’), and ’pregnancy, health.’ A strategy of using wildcard search terms such as ’Behavior, Environment, Internet’ and a Boolean operator was adopted. As a result of the database search, 82 (PubMed), 19 (CINAHL), 10 (Embase), 6 (ERIC), 230 (Cochrane Library), 1 (KISS), and 3 (RISS) studies were found, and 8 articles were found via a hand search. After reading all of the titles and abstracts of the studies and selecting the papers to be referenced in the IEPP, a total of 19 papers, including 17 international papers and 2 papers from South Korea, were reviewed to derive the themes. The researchers derived six core themes: fine dust, EDCs, heavy metals, electromagnetic waves, food, and environmental toxins. The needs of pregnant women in terms of environmental health [11] were found to focus on fine dust, electromagnetic waves, food, and chemical substances.

Second, the revised protection motivation theory (rPMT) [20] was used during the design stage. The main concept of the rPMT is to determine the purpose, content, media, subjects, and duration in order to provide an effective intervention for enhancing individuals’ health perceptions. The educational purpose was to promote positive environmental health perceptions and behaviors among pregnant women using a non-face-to-face program. The content of each of the four sessions included sections on 1) understanding chemical hazards and micro-dust, 2) fetal health problems induced environmental toxin, 3) protection behaviors from EDCs and electromagnetic waves during pregnancy, and 4) environmental health behaviors during postpartum. For the control group, treatment as usual (TAU) was performed as knowledge of pregnancy, delivery, postpartum care, and neonatal care during four weeks through face-to-face education in small group under ten participants at the birth class in the public health center; Zoom video conferencing was selected as a medium to enable two-way audio and video communication in real-time. A group meeting room in KakaoTalk messenger was created to ask questions and share messages of maternal empowerment, encouragement, and social support. Two professors of maternal nursing and one midwife led the education sessions and answered questions. The intervention was conducted across 2-hour sessions and took place every Wednesday for 4 weeks, for a total of 8 hours (Table 1).

**Table 1:** Internet-based environmental prenatal health program via Zoom video conferencing.

Session	Categories	Contents	Duration	Educator
Week 1–1	Prenatal	• Orientation (purpose, content, methods of the program) • Weekly schedule announcement • Health promotion during pregnancy	60 min	Professor 1
Week 1–2	Environmental	• Sharing environmental concerns related to pregnancy • Chemical hazards during pregnancy • Micro-dust reduction behaviors for pregnant women	60 min	Professor 1
Week 2–1	Prenatal	• Childbirth education • Understanding of childbirth • Healthcare management during childbirth	60 min	Midwife
Week 2–2	Prenatal Environmental	• Pain control during childbirth • Practice breathing and relaxation • Environmental toxin and fetal health	30 min 30 min	Midwife
Week 3–1	Environmental	• Learn about water and soil contamination during pregnancy • Reducing exposure to endocrine disruptors and electromagnetic waves	60 min	Professor 2
Week 3–2	Prenatal Environmental	• Prenatal health promotion • Learn about environmental health behaviors • Discussion about environmental health behaviors	30 min 30 min	Professor 2
Week 4–1	Environmental	• Sharing environmental concerns related to newborns • Childcare and breastfeeding while avoiding environmental hazards • Evaluation of the environmental health promotion program	60 min	Professor 1
Week 4–2	Prenatal	• Understanding the postpartum period • Healthcare management during postpartum • Wrap-up of the prenatal class	60 min	Professor 1

Third, in the development stage, the intervention’s feasibility, applicability, and relevance to participants were discussed and revised. PowerPoint materials for professors and pregnant women were created. The content validity was verified by a public health center nurse, an obstetric clinic nurse, and a maternal nursing professor. Based on their feedback, difficult academic terms were deleted, and the materials that focused on daily life were changed. The content validity index (CVI) was checked for appropriateness, feasibility, importance, and adaptability. The CVI was rated using a Likert scale that ranged from 1 point, indicating “very inappropriate,” to 5 points, indicating “very appropriate.” The item-level content validity index (I-CVI) were 1.00 which were higher than the criterion .78 [21]. The scale-level content validity index of usual agreement [S-CVI (ua)] was .90 and the scale-level content validity index averaging [S-CVI (ave)] was 1.00 which were higher than the criterion of .90 [21]. The final content was selected and spread across 8 sessions over 4 weeks via lectures, discussions, and questions and answers.

Fourth, a rehearsal of intervention for consultation was conducted during the implementation stage before the main application of the IEPP. In April 2021, an online lecture for 9 master’s degree students was held for 2 hours through Zoom video conferencing. The researchers discussed the relevance and effectiveness of the content.

Fifth, during the analysis stage, the intervention effects were evaluated in terms of environmental health perceptions, behaviors, and educational satisfaction through a questionnaire survey. We surveyed educational satisfaction in the post-test because the satisfaction was regarding the intervention.

### 2. IEPP application

#### 1) Study design

This was a quasi-experimental study that used a non-equivalent control-group pretest-posttest design. This study investigated the effects of an IEPP using Rogers’ [20] rPMT as a theoretical framework. This study was conducted according to the Transparent Reporting of Evaluations with Non-randomized Designs statement [22].

#### 2) Participants

The researchers recruited pregnant women who were attending antenatal classes at public health centers located in C City of Gangwon Province and G City of Chungcheong Province using the convenience sampling method. Researchers explained the purpose of the study, the data collection period, and the research methods and obtained permission via written consent forms. The inclusion criteria were 1) pregnant women at over four weeks’ gestation and aged 18 or older, 2) women who wanted to participate in the IEPP, 3) women who indicated that they were willing to participate for the full 4 weeks, and 4) women who had a smart gadget. The exclusion criteria were 1) pregnant women who were hospitalized for health problems during pregnancy and 2) pregnant women who could not read Korean.

**Fig 1:**
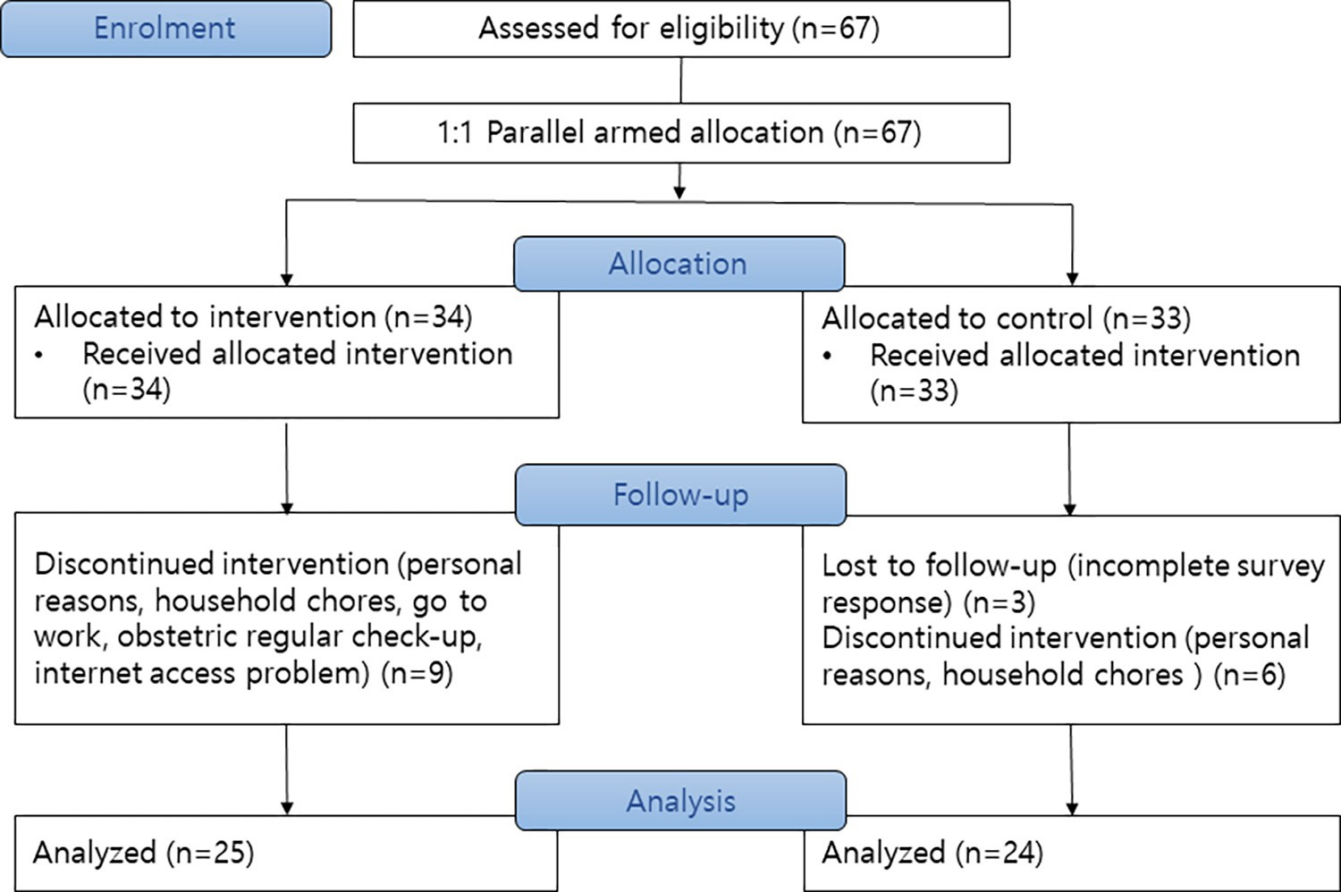
Flow diagram of the research process.

The sample size was determined using G*Power 3.1.0 with the one-tailed in dependent sample t-test, an effect size (f) of 0.50, power of 0.80, a significance level of 0.05 with a two-sided test, and 1:1 parallel-arm assignment, resulting in a total of 21 people in each group [23]. The power was set with a high effect size by calculating Cohen’s d [24]. Given a possible dropout rate of 10%, a total of 50 people were recruited, with 25 subjects in each group. In the pre-test, the experimental group started including 34 people enrolled in the study, and the control group including 33 people. In the posttest, final participants were included 25 out of 34 subjects from the experimental group and 24 out of 33 subjects from the control group, totaling 49 participants. The unit of assignment was individuals, and all participants in the class were included to minimize the potential bias resulting from non-randomization. The enrollment and intervention were independently performed apart region and the participants did not know whether they were included experimental or control group. The participants were single blinded to the intervention even when researchers were not blinded to the assignment conditions. Based on the preliminary survey, the retention rate was 73.5% in the experimental group and 72.7% in the control group. The reasons for dropout included non-completion of the training in the experimental group (n = 9), non-completion of the training in the control group (n = 6), and failure to respond to the online posttest (n = 3). The drop-out rates and reasons were similar in the both group. The drop out reasons were personal reasons, household chores, job, obstetric regular check, and internet access problem in the experimental group and personal reasons, household chores, and incomplete post-test survey in the control group (Fig 1). Data were collected from 21 April 2021 to 24 November 2022.

#### 3) Measurements

*(1) Environmental health perceptions*: *Primary outcomes*. The women’s environmental health perception scales [25] were used with permission. These scales are based `on Rogers’ [20] rPMT as a theoretical framework to measure severity, susceptibility, response efficacy, self-efficacy, benefit, and barriers in terms of environmental health. The severity scale includes 10 items across 3 subscales: 4 items on “chemicals,” 3 items on “electromagnetic waves,” and 3 items on “harmful foods.” The susceptibility scale includes 11 items across 2 subscales: 7 items on “reproductive health” and 4 items for “general health.” Response efficacy includes 10 items across 2 subscales: 7 items on “avoiding toxicants” and 3 items on “pursuit of health.” Self-efficacy includes 14 items across 3 subscales: 5 items on “prevention efficacy,” 5 items on “judgment efficacy,” and 4 items on “control efficacy.” The benefits scale includes 8 items across 2 subscales: 5 items on “psychological benefits” and 3 items on “physical benefits.” The barriers scale includes 10 items across 2 subscales: 5 items on “negative atmosphere” and 5 items on “burden.” Answers are provided using a 5-point Likert scale, ranging from 1 point, meaning “not at all,” to 5 points, meaning “strongly agree.” The reliability in the original study and the present study, as indicated by Cronbach’s alpha, were .84 and .83 for severity, .92 and .95 for vulnerability, .88 and .92 for response efficacy, .90 and .86 for self-efficacy, .91 and .85 for benefits, and .85 and .74 for barriers, respectively.

*(2) Environmental health behaviors*: *Primary outcomes*. The women’s environmental health behavior scales [25] were used with permission. The environmental health behavior scales measure personal and community health behaviors based on Rogers’ [20] rPMT. Personal health behaviors include 14 items across 4 subscales: 6 items on “lifestyle,” 4 items on “personal goods,” 3 items on “food,” and 3 items on “fine dust.” Community health behaviors include 16 items across 4 subscales: 5 items on “reduction,” 5 items on “involvement,” 3 items on “recycling,” and 3 items on “reuse.” Answers are provided using a 5-point Likert scale, with possible scores ranging from 1 point, meaning "not at all," to 5 points, meaning "strongly agree." Total possible scores range from 17 to 85 points for personal health behaviors and 16 to 80 points for community health behaviors. A higher score indicates more environmental health behaviors. The reliability in the original study and the present study, as indicated by Cronbach’s alpha, were .90 and .88 for personal health behaviors and .91 and .90 for community health behaviors, respectively.

*(3) Education satisfaction*: *Secondary outcomes*. Education satisfaction was measured using the curriculum effectiveness scale [12], with permission, based on a study on educational satisfaction [26]. The curriculum effectiveness scale consists of 3 items: feasibility, accessibility, and overall satisfaction. Answers are provided using a 10-point Likert scale, ranging from 1 point, meaning “very difficult/very uncomfortable/very dissatisfied,” to 10 points, meaning “very easy/very comfortable/very satisfied,” and total possible scores range from 3 to 30 points. The reliability, as indicated by Cronbach’s alpha, was .95 in the original study and .82 in this study.

#### 4) Data collection

Data collection was requested by the heads of the health centers and the maternal and child health teams at 2 public health centers. Recruitment was conducted via websites, brochures, and social network services at two public health centers. The experimental group was recruited at C city and applicants met criterions assigned to attend an online antenatal class for a total of four sessions, and in April, July, September, and November 2021, four 2-hour education sessions were conducted once per week via Zoom video conferencing. The experimental group participated in a KakaoTalk group chat room for question and answer communication. The control group was recruited at G city and all applicants met criterions were assigned to attend a face-to-face prenatal class for a total of four sessions, and in May, July, September, and November 2021, four 2-hour training sessions were conducted in the education room at the public health center. For the experimental group, the researcher conducted an online survey, and for the control group, the researcher conducted a face-to-face survey while following quarantine rules related to the COVID-19 pandemic. The post-study survey was conducted in the same way immediately after the end of the four sessions. For those in the control group who completed the follow-up survey, additional printed educational materials related to environmental health behaviors were provided and questions were answered. The participants were provided with an online gift at the pretest and posttest stages.

#### 5) Research procedure

*(1) Step 1*: *Preliminary test*. A pilot test was conducted to confirm the suitability of the questionnaire and to address problems with the educational materials. The program was conducted at a public health center in G City, and educational materials were presented to six pregnant women aged from 22 to 30 at a prenatal class in April 2021. The effects of environmental health behaviors in daily life on fetuses were emphasized. The questionnaire was not modified and took approximately 20 minutes to complete. Repetition in the online questionnaire increased the participants’ fatigue, so the questionnaire was modified to increase readability.

*(2) Step 2*: *Pretest*. The experimental group completed an online questionnaire survey during a Zoom meeting at the public health center in C City, and the control group completed a face-to-face questionnaire survey at the public health center in G City. The pretest collected the participants’ general characteristics, obstetric characteristics, environmental health perceptions, and behaviors.

*(3) Step 3*: *Implementation of the IEPP*. For the experimental group, the prenatal education program was conducted by researchers and midwives via Zoom video conferencing in April, July, September, and November 2021. For the control group, a standard face-to-face prenatal education program was conducted in the public health center in May, July, September, and November 2021. The content for the control group included sections on understanding pregnancy, antenatal care, prenatal health problems, the birth process, pain control, labor breathing, postpartum care, breastfeeding, and infant care. After each education session, social support was provided through question-and-answer sessions, and the participants were encouraged to share their opinions in both groups.

*(4) Step 4*: *Posttest*. The posttest was conducted immediately after the completion of the 4-week prenatal education program. A section was added to the questionnaire on education satisfaction in addition to the items included in the pretest questionnaire. The posttest was completed by the control group at the public health center and through an online survey by the experimental group.

#### 6) Data analysis

The collected data were analyzed using SPSS/WIN 26.0 (IBM Corp., Armonk, NY, USA). The subjects’ general characteristics, obstetric characteristics, environmental health perceptions, behaviors, and educational satisfaction were analyzed using the frequency, percentage, mean, and standard deviation. The t-test and chi-square test were used to test the homogeneity of the characteristics of the control and experimental groups. The Shapiro-Wilk test was used to test the normality of variables, and the ANCOVA (Analysis of Covariance) and t-test were performed to test the difference in posttest variables between the control and experimental groups. An intention-to-treat analysis was not applied, and non-compliers were excluded in the final analysis.

### 3. Ethical considerations

The institutional review board of the university approved this study (KUB-IRB-2021-04). After obtaining permission from the heads of the public health centers, the researcher conducted the survey. In order to protect the rights of the participant, informed consent documentations with participants’ signatures were obtained after the purpose and methods of the study, the benefits and drawbacks of participation in the study, privacy and confidentiality, and the possibility of refusal and withdrawal from the study before data collection were explained. There was no culturally minority or under 18 aged participants. It was registered at Clinical Research Information Service of the Republic of Korea (KCT0007086). The authors confirm that all ongoing and related trials for this intervention are registered.

## Results

### 1. Subject homogeneity test

The age, gestational age, number of children, employment status, morbidity status, environmental health perceptions, and behaviors were homogenous in both groups. In the control group and experimental group, respectively, the mean age was 32.96±4.58 and 35.28±4.27 (t = -1.83, *p* = .073), the mean gestational age was 23.54±8.53 and 23.56±7.62 weeks (t = -0.01, *p* = .994), the mean number of children was 0.58±0.97 and 0.20±0.41 (t = 1.78, *p* = .085), and the number of participants who were employed was 6 and 12 (χ^2^ = 3.76, *p* = .052). The number of participants with morbidities was 3 (hyperthyroidism, cystitis, anemia) in the control group and 3 (tendonitis, diabetes, ankle pain) in the experimental group (*p* = .957). The mean scores for environmental health perceptions in the control and experimental groups, respectively, were 38.00±7.63 and 41.24±4.15 (t = -0.85, *p* = .070) for severity, 42.42±2.99 and 44.68±5.05 (t = -1.89, *p* = .064) for susceptibility, 40.50±4.70 and 42.88±3.85 (t = -1.94, *p* = .060) for response efficacy, 44.42±6.31 and 44.20±6.47 (t = 0.12, *p* = .906) for self-efficacy, 28.88±5.26 and 30.48±4.26 (t = -1.17, *p* = .246) for benefits, and 32.04±5.63 and 31.52±4.76 (t = 0.35, *p* = .728) for barriers. The mean scores for environmental health behaviors in the control and experimental groups, respectively, were 59.75±8.47 and 58.28±8.28 (t = 0.62, *p* = .540) for personal environmental health behaviors and 56.42±9.19 and 56.96±8.94 (t = 0.18, p = .861) for community environmental health behaviors (Table 2).

**Table 2:** Analysis of homogeneity between the experimental and control groups (N = 49).

Characteristics	Control group (n = 24)		Experimental group (n = 25)	t/χ^2^	*p*
	n (%) / M (SD)		n (%) / M (SD)		
Age (years)		32.96 (4.58)	35.28 (4.27)	-1.83	.073
Gestational age (weeks)		23.54 (8.53)	23.56 (7.62)	-0.01	.994
Number of children		0.58 (0.97)	0.20 (0.41)	1.78	.085
Employment status	Yes	6 (25.5)	12(48.0)	3.76	.052
	No	18 (75.0)	13(52.0)		
Morbidity status	Yes†	3(12.5)	3(12.0)	0.01	.957§
	No	21(87.5)	22(88.0)		
Environmental severity		38.00 (7.63)	41.24 (4.15)	-1.85	.070
Environmental susceptibility		42.42 (2.99)	44.68 (5.05)	-1.89	.064
Environmental response efficacy		40.50 (4.70)	42.88 (3.85)	-1.94	.060
Environmental self-efficacy		44.42 (6.31)	44.20 (6.47)	0.12	.906
Environmental benefits		28.88 (5.26)	30.48 (4.26)	-1.17	.246
Environmental barriers		32.04 (5.63)	31.52 (4.76)	0.35	.728
Personal environmental behaviors		59.75 (8.47)	58.28 (8.28)	0.62	.540
Community environmental behaviors		56.42 (9.19)	56.96 (8.94)	0.18	.861

§Fisher’s exact test

†Hyperthyroidism, cystitis, anemia, tenosynovitis, gestational diabetes, ankle fracture

M, mean; SD, standard deviation.

### 2. Efficacy test of the IEPP

As a result of the Shapiro-Wilk normality test of the dependent variable and Levene’s test for the equality of variance test, the *p*-values were found to satisfy the assumption of the ANCOVA.

#### 1) Primary outcome effects of the IEPP on environmental health perceptions

Hypothesis 1 was partially supported. The severity score was significantly higher in the IEPP group than in the control group (40.92±3.93 and 45.76±3.47) (F = 17.96, *p* < .001). The response efficacy score was significantly higher in the IEPP group than in the control group (40.25±5.40 and 45.88±4.42) (F = 15.69, *p* < .001). In addition, the total environmental perceptions score was significantly in the IEPP group than in the control group (236.83±22.80 and 250.68±15.43) (F = 7.80, *p* = .008). There were no statistically significant differences with regard to susceptibility (43.92±4.74 and 45.92±4.46) (F = 2.09, *p* = .155), self-efficacy (45.67±8.76 and 47.80±6.72) (F = 0.62, *p* = .435), benefits (30.04±4.81 and 32.92±4.04) (F = 2.82, p = .054), and barriers (32.21±4.32 and 32.40±4.88) (F = 0.11, *p* = .741) between the control and experimental groups (Table 3).

**Table 3:** Effects of the program on environmental health perceptions and behaviors between groups (N = 49).

Variables		Control group (n = 24)	Experimental group (n = 25)	F^§^	*p*
		M (SD)	M (SD)		
Environmental severity	Pretest	38.00 (7.63)	41.24 (4.15)	17.96	< .001
	Posttest	40.92 (3.93)	45.76 (3.47)		
Environmental susceptibility	Pretest	41.29 (4.51)	44.68 (5.05)	2.09	.155
	Posttest	43.92 (4.74)	45.92 (4.46)		
Environmental response efficacy	Pretest	40.50 (4.70)	42.88 (3.85)	15.69	< .001
	Posttest	40.25 (5.40)	45.88 (4.42)		
Environmental self-efficacy	Pretest	44.42 (6.31)	44.20 (6.47)	0.62	.435
	Posttest	45.67 (8.76)	47.80 (6.72)		
Environmental benefits	Pretest	28.88 (5.26)	30.48 (4.26)	2.82	.054
	Posttest	30.04 (4.81)	32.92 (4.04)		
Environmental barriers	Pretest	32.04 (5.63)	31.52 (4.76)	0.11	.741
	Posttest	32.21 (4.32)	32.40 (4.88)		
*Total environmental perceptions*	Pretest	225.03 (18.15)	235.00 (3.51)	7.80	.008
	Posttest	236.83 (22.80)	250.68 (15.43)		
Personal environmental behaviors	Pretest	59.75 (8.47)	58.28 (8.28)	0.34	.562
	Posttest	58.79 (7.80)	61.32 (8.29)		
Community environmental behaviors	Pretest	56.42 (9.19)	56.96 (8.94)	0.91	.344
	Posttest	58.25 (9.26)	57.00 (7.50)		
*Total environmental behaviors*	Pretest	119.67 (13.56)	118.32 (14.10)	0.05	.815
	Posttest	119.67 (13.56)	118.32 (14.10)		

M, mean; SD, standard deviation.

§The F score was derived from an ANCOVA with age, number of children, and dummy variables of employment status.

#### 2) Primary outcome effects of the IEPP on environmental health behaviors

Hypothesis 2 was rejected. There were no statistically significant differences in personal health behaviors (58.79±7.80 and 61.32±8.29) (F = 0.34, *p* = .562), community health behaviors (58.25±9.26 and 57.00±7.50) (F = 0.91, *p* = .344), or the total score (119.67±13.56 and 118.32±14.10) (F = 0.05, *p* = .815) between the control and experimental groups (Table 3).

#### 3) Secondary outcome effects of IEPP educational satisfaction

Hypothesis 3 was rejected. There were no statistically significant differences in feasibility (7.71±2.19 and 8.48±1.81) (t = -1.34 *p* = .185), accessibility (8.42±1.16 and 8.68±1.95) (t = -0.41, *p* = .677), overall satisfaction (8.42±1.76 and 8.68±1.95) (t = 0.51, *p* = .608), and the total score (24.83±5.21 and 25.56±4.09) (t = -0.50, *p* = .618) between the control and experimental groups (Table 4).

**Table 4:** Differences in educational satisfaction between groups (N = 49).

Variables	Control group (n = 24)	Experimental group (n = 25)	t/χ^2^	*p*
	M (SD)	M (SD)		
Feasibility	7.71 (2.19)	8.48 (1.81)	-1.34	.185
Accessibility	8.42 (1.16)	8.68 (1.95)	-0.41	.677
Satisfaction	8.42 (1.76)	8.68 (1.95)	0.51	.608
*Total*	24.83 (5.21)	25.56 (4.09)	-0.50	.618

M, mean; SD, standard deviation.

## Discussion

This study developed, implemented, and evaluated the effect of an internet-based prenatal education program on environmental health perceptions, behaviors, and educational satisfaction based on rPMT [20]. Pregnant women who attended the IEPP had stronger environmental perceptions than those who attended a face-to-face general prenatal education program, and the experimental group’s severity, response efficacy, and total environmental perception of environmental health increased. In terms of environmental health behaviors and educational satisfaction, the IEPP did not have a statistically significant differences. Rogers [20] explained health behaviors were motivated through perception of the benefit and barriers in the internal process of severity, susceptibility, response efficacy, and self-efficacy in advance [20]. The results of this study showed the environmental behaviors were not fully motivated but participants adopted the emotional factors and behavioral appraisal related environmental threat.

The findings related to the severity of environmental health problems in this study reflect the common fear of environmental toxins considered to be harmful during pregnancy and the intensity of the danger [26]. Evidence of negative effects on the health of both fetuses has shown that environmental toxins can lead to delays in neurological and physical development in infancy and early childhood [27]. Studies of environmental toxins such as electromagnetic waves, radiation, heavy metals, and micro-dust have also found negative health outcomes on pregnant women and fetuses. These risks should be addressed in prenatal education programs [28]. In this study, given that the participants’ awareness of the severity of environmental health issues increased after the IEPP, an internet-based prenatal education program could inspire motivation.

Response efficacy refers to the degree to which pregnant women believe that behavior will help maternal and fetal health [26]. There have been studies in which programs to reduce pregnant women’s exposure to EDCs were found to affect children’s physiological indicators [29]. In this study, the internet-based intervention played an important role in increasing the participants’ positive environmental health perceptions.

Benefits are gained from environmental health behaviors as a reward for an adaptive response [26]. If a pregnant woman consumes fewer unhealthy foods, she may experience physical benefits such as better digestion and psychological comfort [30]. If the attractiveness of the reward is greater than the sum of severity and vulnerability, environmental behavior is more likely to be selected. Otherwise, compensation and benefits were not effective to motivate found environmental behaviors [30]. In this study, the perceived benefits of positive environmental health behaviors not statistical significant increased after the IEPP. Therefore, birth educators would be better to empower strategies to increase the attractiveness of rewards by emphasizing the benefits of positive environmental health behaviors.

Susceptibility refers to the degree to which a person believes health problems may occur due to environmental toxins during pregnancy [25]. Susceptibility similar with vulnerability refers to participant’s perception of the likelihood that health problems will affect herself. In the health beliefs model, participants interpret susceptibility as mental paths, maximizing behavioral outcomes in a way that corresponds to their values and expectations [20]. Pregnant women often experience anxiety about the health of their fetuses due to their vulnerability to environmental risks. Pregnancy-related health problems are possible even with little exposure to environmental toxins since pregnancy is considered a vulnerable period [31]. Since pregnancy affects women’s families and the next generation, preventing exposure to hazardous substances is particularly important [31]. It is necessary to devise online education programs that feature a stronger emphasis on pregnant women’s vulnerability since no statistically significant effect was observed in this study.

Self-efficacy refers to the integrated perception of a person’s belief and confidence in his or her abilities [31]. According to cognitive psychology, the processes that lead to changes in knowledge, beliefs, thoughts, intentions, and actions are complex. Further research is needed to understand pregnant women’s perceptions of coping appraisal and motivations, and the cognitive processes of pregnant women should be assessed [32].

Personal behaviors refer to health behaviors that prevent exposure to environmental toxins and protect the mother and fetus from environmental health problems [31]. Evidence on the effects of environmental toxins on the health of pregnant women and fetuses has recently accumulated [28]. Longitudinal studies found that exposure to chemicals caused abnormal levels of maternal hormones and reproductive health problems [33] and electromagnetic waves in pregnant women was associated with miscarriage before 12 gestational weeks [5]. Personal health behaviors of pregnant women ultimately affect the health of their children, including when they are still embryos, fetuses, and recent newborns [34]. In this study, given that the differences between the groups related to personal behaviors after the IEPP were not significant, an approach to motivate changes in behavior is needed.

Community behavior refers to alternative actions to bring about changes in lifestyle that are altruistic and less burdensome on the environment [25]. The environment cannot be separated from individual health, and the environment can be thought of as an ecological concept that coexists with pregnancy. Therefore, community health behaviors are directly related to the environment and inspire the next generation’s behaviors [35]. In this study, since community behaviors in pregnant women were found not to have increased after the IEPP, techniques such as network, group therapy, community of practice, and train-to-trainer approach should be incorporated in future interventions [36].

In this study, there were no significant differences between the groups in terms of feasibility, accessibility, and satisfaction. One disadvantage of non-face-to-face education is the lower degree of cohesion compared to face-to-face education, and the ongoing participation rate in online programs tends to gradually decrease over time. In addition, only learners with access to the internet can attend online education programs [18]. However, the internet-based prenatal education program in this study did not show lower satisfaction compared to the face-to-face program, and it can still be used as a viable alternative to face-to-face education.

This study had several limitations. Since this was a quasi-experimental study conducted at two public health centers in South Korea, the sample was relatively small, and the findings cannot be generalized. In addition, the study was not conducted using double-blind and random sampling. Recruitment for the face-to-face education program was also difficult due to the COVID-19 pandemic, and the study was limited since the data collection period was long due to the requirement to include fewer than 10 participants per session to maintain social distancing. The gestational age of participants was varied from first to third trimester even though the environmental toxin was more harmful in the early gestation because of the vulnerability of the embryo. We suggest the environmental intervention adopted in the first trimester pregnant women. Future studies are needed to verify the effectiveness of internet-based interventions and diversify the types of educational interventions, including online media, mobile applications, counseling, and mentoring, and regional expansion should be promoted to include a larger proportion of pregnant women.

## Conclusion

This study aimed to test the effect of an internet-based environmental prenatal health education program for pregnant women based on Rogers’ rPMT and conducted via Zoom video conferencing [20]. An environmental prenatal health education program was developed and conducted at two public health centers from April to November 2021, and the results of the pretest-posttest were collected for control and experimental groups. The IEPP developed in the study led to stronger environmental perceptions than the face-to-face program that included only general prenatal education. The severity, response efficacy, and total environmental perception of environmental health improved more in the experimental group than in the control group. Environmental health behaviors and educational satisfaction did not significantly differ between the face-to-face and internet-based programs. Therefore, the implementation of the internet-based environmental prenatal health education program is recommended since it can be applied in the context of the COVID-19 pandemic. We suggest that offering smart gadgets to vulnerable population to provide equal opportunities in the pandemic era. The internet-based education can be extended to family participatory program with husband of pregnant women because of merits space and time. Environmental pollution is worsening globally and the health problems experienced by pregnant women and fetuses caused by environmental toxins are more common. Therefore, the results of this study can improve the quality of healthcare and lead to the development of internet-based programs at public health centers, hospitals, and institutions.

## Supporting information

S1 Checklist TREND statement checklist. (PDF)

S1 Dataset Raw response data from participants. (XLSX)

S1 File (DOCX)
