# Supplementary material for: The effects of environmental prenatal program on environmental health perception and behavior using internet-based intervention in South Korea: A non-randomized controlled study
Source: PLoS One. 2022 Nov 15;17(11):e0277501. doi: 10.1371/journal.pone.0277501 (PMC9665389; doi:10.1371/journal.pone.0277501)
Supplement: S1 File — (DOCX) [file pone.0277501.s003.docx]

**Study protocol**

**Title:** Impact of Internet-based Educational Intervention for Environmental Health Behavior among Pregnant Women

**1. Background**

Climate change and environmental pollution exacerbate the effects of environmental toxicants on human health. Environmental toxins refer to chemicals, heavy metals, electromagnetic waves, and radiation that harm the human body via water, food, air, and soil [1]. In particular, women are sensitive to environmental toxins due to their higher number of hormone receptors with which environmental toxins are capable of interacting. Women are especially vulnerable to environmental toxins during pregnancy, and environmental toxins absorbed through the placenta can cause fetal problems [2]. Pregnancy is a critical window in a woman’s life cycle regarding exposure to environmental toxins [1].

The effects of environmental toxins on pregnant women and fetuses have been studied in recent years, including endocrine disruptors (EDCs) on pregnant women and fetuses. The rate of preterm births was 1.6 times higher among women who consumed drinking water contaminated with parabens and bisphenol A [3]. The consumption of lead-contaminated water lowered the overall fertility rate and increased the number of low-birthweight infants [4]. During early pregnancy, exposure to electromagnetic waves was associated with increased spontaneous abortion [5]. In fetuses, EDCs caused psychomotor development and reproductive health [6]. Bisphenol A, phthalate, and pesticide exposure decreased the number of primordial follicles in fetuses [7]. As fetuses grow, phthalates shorten the fetal femur and reduce the weight of newborns and children aged 2 to 5 years [8]. Exposure to pesticides during pregnancy slowed children’s cognitive development [9].

Healthcare providers are responsible for providing education to prevent environmental health problems in pregnant women and fetuses [2]. Traditionally, South Korea has a prenatal culture where prenatal education (taegyo) protects the mother and fetus from environmental toxins [10]. Currently, antenatal education is conducted at public health centers, hospitals, and private institutions. Existing prenatal education programs focus on understanding physiological changes, childbirth, postpartum care, and newborn care. Few prenatal education programs include relevant information to protect mothers and fetuses from environmental toxins [11]. As a study on environmental health behavior interventions for pregnant women reported, environmental interventions that used kiosks [12] were accompanied by increases in environmental knowledge, perception, and behavior.

Due to the coronavirus disease 2019 (COVID-19) pandemic, which has continued for more than 2 years, social distancing has become mandatory, and few face-to-face education programs have been conducted [13]. Internet-based education interventions, a representative method of non-face-to-face education, refer to interventions in which educators and learners develop and share educational content via the internet [14]. There are various methods of conducting internet-based educational interventions for pregnant women, including web-based interventions, which constitute a method for learners to access educational programs through websites [15]. Mobile phone applications for health purposes have grown [16].

Pregnant women are sensitive to environmental pollution and need environmental health education to protect themselves from environmental risk factors [11]. Therefore, this study developed and investigated the effects of an internet-based intervention to promote positive environmental health behaviors in pregnant women.

**2. Objectives**

This study aimed to confirm that an environmental prenatal education program using an internet-based intervention affected pregnant women’s environmental health perceptions and behaviors. The specific research objectives were as follows: first, to develop an internet-based environmental prenatal program (IEPP) for pregnant women; second, to investigate the effect of an IEPP on environmental health perceptions and behaviors; and third, to investigate the effects of an IEPP on educational satisfaction.

**3. Researchers**

| Position | Name | Affiliation | status | Role |
| --- | --- | --- | --- | --- |
| Principal investigator | Hyun Kyoung Kim | ^1^Department of Nursing, Kongju National University | Assistant professor | Principal investigator |
| Researcher | Geum Hee Jeong | School of Nursing, Hallym University | Professor | Corresponding author |
| Researcher | Hye Young Min | College of Nursing, Ewha Woman’s University | Ph.D. student | Co-author |

**4. Funding organization**

| Funder | Content | Comment |
| --- | --- | --- |
| National Research Foundation of Korea | Research project |  |

**5. Study period**

Estimated study period: One year From the date of IRB approval

Survey study period: One year From the date of IRB approval

**6. Study subjects**

- Inclusion criteria: 1) pregnant women at over four weeks’ gestation and aged 18 or older, 2) women who wanted to participate in the IEPP, 3) women who participated for the entire 4 weeks, and 4) women who had an intelligent gadget.

-Exclusion criteria: 1) pregnant women hospitalized for health problems during pregnancy and 2) pregnant women who could not read Korean.

- Evidence for selecting the subject was based on the systemic review as follows: Ashford, M. T., Olander, E. K., & Ayers, S. (2016). Computer- or web-based interventions for perinatal mental health: A systematic review. Journal of affective disorders, 197, 134–146. http://doi.org.ssl.access.ewha.ac.kr/10.1016/j.jad.2016.02.057

**7. Study size estimation**

The sample size was determined using G*Power 3.1.0 with the two-tailed independent sample t-test, an effect size (f) of 0.85, power of 0.80, a significance level of 0.05 with a two-sided test, and 1:1 parallel-arm assignment, resulting in a total of 23 people in each group. The effect size and the power were calculated based on a similar previous study: Pell, T., Eliot, M., Chen, A., Lanphear, B. P., Yolton, K., Sathyanarayana, S., & Braun, J. M. (2017). Parental concern about environmental chemical exposures and children’s urinary concentrations of phthalates and phenols. *The Journal of Pediatrics, 186*, 138-144. http://dx.doi.org/10.1016/j.jpeds.2017.03.064

G*Power 3.1 was available from Faul, F., Erdfelder, E., Buchner. A., & Lang, A. (2009). Statistical power analyses using G*Power 3.1: Tests for correlation and regression analyses. *Behavior Research Methods, 41*(4), 1149-1160. <https://doi.org/10.3758/BRM.41.4.1149>

**8. Recruitment of participants**

The self-report questionnaire will be filled out by subjects recruited through random sampling. Only subjects who have signed the informed consent can participate in the survey. The purpose of the study, contents, report of results, cancellation during the survey, and protection of confidentiality will be fully explained. The places of subject recruitment by random sampling are Gongju-si, Chungcheongnam-do, and Chuncheon-si, Gangwon-do. After seeking cooperation from the head of the public health center and the head of the maternal and child health team, the recruitment of participants is requested. The researcher explains the purpose of the study to the subject. She collects data only for those who consent to participate in this study. Expressly, a research guide for pregnant women visiting public health centers is provided to public health centers for recruitment. The researcher receives the subject’s phone number, who has agreed to participate in this study, directly from the subject. The purpose, content, report of the study results, the possibility of cancellation during the survey, and the protection of confidentiality are fully explained over the phone, and a survey is conducted to pregnant women who have consented.

**9. Informed consent**

After explaining the purpose of the study, the informed consent form must be written by subjects face-to-face. In the consent form, the followings are specifically described: research purpose, research content, survey duration, gain and risk due to the survey, the possibility of withdrawal and rejection any time, privacy protection, confidentiality, research results utilization method, research data use, storage method, time limit of original data, and disposal of data. The researcher explains the content of the informed consent form. Subjects’ consent is confirmed with a handwritten signature and the researcher’s signature.

**10. Methods**

-Curriculum content development: The curriculum development theory utilizes the ADDIE (Analysis, Design, Development, Implementation, Evaluation) instructional design model. It is developed through the analysis, design, development, execution, and evaluation stages. In the analysis stage, a topic is derived through a systematic literature review related to the environmental health of pregnant women.

- Design stage: Create a draft of an internet-based curriculum using video, YouTube, and Kakao Talk as a curriculum medium to deliver the topic derived from the analysis stage.

- Development Stage 1: Produce a video based on the draft of the curriculum media development.

- Development Stage 2: Curriculum validity test: Content validity is also verified by 3 women’s health experts (the head of the maternal and child health team at the public health center, maternal and child health nutritionist, and one professor in women’s health nursing). A 5-point Likert scale is used to ask the validity of the content, and if it is 0.78 or higher, it is adopted and is corrected through personal advice.

-Reference: Polit DF, Beck CT, Owen SV. Is the CVI an acceptable indicator of content validity? Appraisal and recommendations. Research in Nursing & Health. 2007;30(4):459-467.

-Asurvey is conducted in advance to provide Internet-based education to pregnant women in the control and experimental groups.

- The control group participates in face-to-face general childbirth education (pregnancy management, breastfeeding, postpartum management, newborn care) at public health centers: 4 times (2 hours per session, a total of 8 hours, once a week, face-to-face general childbirth education)

-A video using KakaoTalk and YouTube social network service is provided to the experimental group (one content per week for 4 weeks): 4 times (2 hours per session for a total of 8 hours, 1 non-face-to-face video or Zoom meeting video training)

- Immediately after the experimental group intervention is completed, the control and experimental groups are given the first post-survey survey.

- A second post-survey survey is conducted on the control and experimental groups to determine the continuity of the educational effect 4 weeks after the experimental group intervention.

- After completing the intervention, the control group also provides the YouTube video address.

- Pre- and post-survey methods: The time required to fill out the questionnaire is within 10 to 15 minutes, and the researcher is responsible for explaining all the research plans and procedures. The research method should be fully explained, and only those who have consented will be used. The pre-survey is conducted face-to-face, and the second post-survey is conducted online using the Naver form.

- Online survey items:

1) General background: age, occupation, gestational age, number of children

2) Health status: Current health problems

3) Curriculum effectiveness: educational feasibility, acceptability, and satisfaction

Source of Measurement tool: O’Mahen HA, Woodford J, McGinley J, Warren FC, Richards DA, Lynch TR, et al. Internet-based behavioral activation-treatment for postnatal depression (Netmums): a randomized controlled trial. Journal of Affective Disorders. 2013;150(3):814-822. http://doi.org/10.1016/j.jad.2013.03.005

4) Environmental health perception (perceived sensitivity, perceived severity, perceived reactive efficacy, perceived self-efficacy, perceived reward, perceived cost, intention to act)

Source of measurement tool: Kim H. K. (2020). Development and assessment of an instrument measuring environmental health perception and behavior toward reproductive health of female adolescents. Japan Journal of Nursing Science, 17(3), e12347.

5) Environmental health behavior (individual health behavior, community health behavior)

Source of measurement tool: Kim H. K. (2020). Development and assessment of an instrument measuring environmental health perception and behavior toward reproductive health of female adolescents. Japan Journal of Nursing Science, 17(3), e12347.

1. **The rationale for the allocation of variables**

Fear of environmental threats is an individual’s assessment of perceived sensitivity, severity, responsiveness, self-efficacy, rewards, and costs. According to the behavioral psychology science theory, it is suitable to explain the psychological mechanism of pregnant women’s environmental health behavior of pregnant women. The intention of behavior is generated, and changes in individual and community health behavior occur [Figure 1].


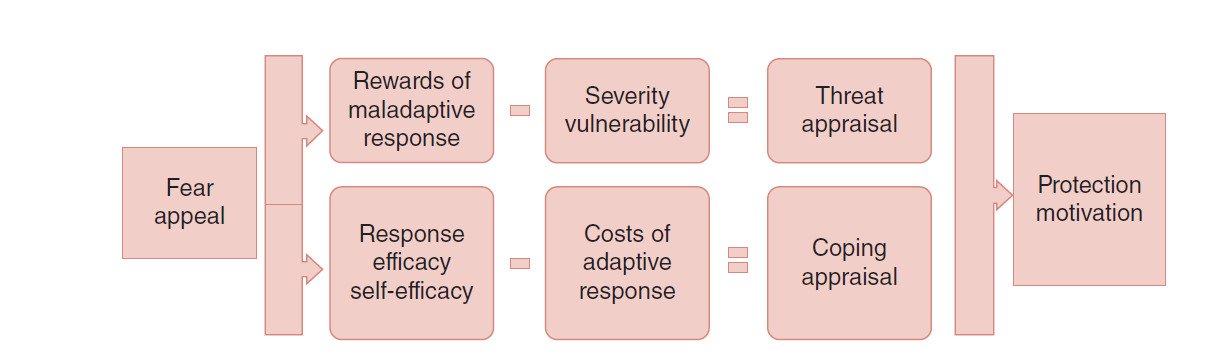


[Figure 1] Environmental Health Curriculum Outcome Variables for Pregnant Women Based on Rogers’ Protective Motivation Theory ( Source of model: Rogers, R.W. (1983). Cognitive and physiological processes in attitude change: A revised theory of protection motivation. In J. T. Cacioppo (Eds.), *Social psychophysiology: A sourcebook* (pp. 153–176). New York, NY: Guilford Press).

**11. Statistical methods**

The collected data is coded in Excel, and quantitative data is analyzed with SPSS 25.0 version statistics program, and the analysis method is as follows.

1) Test for homogeneity of subjects’ general information and health status is analyzed using the actual number, percentage, mean, standard deviation, and independent t-test.

2) The educational process effect difference between the control and experimental groups is analyzed with Repeated Measure ANOVA.

3) The control and experimental groups’ environmental health perception and environmental health behaviors were analyzed with Repeated Measure ANOVA.

**12. Estimated side-effect of the intervention or cautions, and resolution**

In this study, there is no contact with the subject during the development stage of the curriculum. Still, when verifying the effectiveness of the curriculum, a face-to-face meeting is conducted to explain the research and obtain consent from the pregnant woman. Therefore, due to concerns about the spread of COVID-19 to the vulnerable population, the researcher will visit pregnant women during the break time of the childbirth class and follow the quarantine rules while explaining the study and writing a consent form. In addition, childbirth education will be conducted as TAU (Treatment as usual), which is a general childbirth education. The experimental intervention will be conducted through KakaoTalk and YouTube video, a social network service, an initial questionnaire, an educational video of the fourth week, and first and second follow-up questionnaires. Therefore, face-to-face contact with the subject will not occur. In addition, since this study is not an experimental study involving the treatment of human origins, there are no side effects and precautions due to the study. However, consistent research rules by the responsible researcher are required to protect the participants’ privacy.

**13. Criteria of cessation or drop-out**

It includes those who feel burdened with providing questionnaires or videos and want to quit halfway through, those who do not agree to the research, those who express their intention to leave halfway because they feel tired from the survey, and those who have communication difficulties.

**14. Benefit or risk of participants**

Participation in the study may result in time loss of the subject. A small gift is provided to pregnant women worth 6,000 won when they start participating in the study. A small gift worth 18,000 won is an incentive to pregnant women who participated without being eliminated until the second follow-up investigation.

**15. Counter-plan for participants’ safety and protection of personal information**

The data decided to be used in the study minimizes the subject’s personal information. Collected personal information is not provided to third parties. Posting personal information in any media such as the website bulletin board is prohibited. Messenger conducts only individual contact and does not use group chat rooms. Therefore, the possibility of personal information exposure is excluded. The researcher himself conducts the messenger. Personal information collected from research subjects includes age, health status, and obstetrical information. Data are encoded in Excel and stored on the principal researcher’s computer. The computer sets a personal login ID and a password. Personal information data will be destroyed 3 years after the end of the study. The questionnaire papers, the original data are stored in a lockable bookshelf in the researcher’s laboratory. It will be stored for up to 3 years after completion of research, crushed, and discarded. The survey coding is saved by assigning a password. The storage device is also password-protected, so only researchers can access it. Data is permanently deleted from the researcher’s personal laptop computer so that it cannot be restored.

**16. References**

1. Renzo GCD, Conry JA, Blake J, DeFrancesco MS, et al. International Federation of Gynecology and Obstetrics opinion on reproductive health impacts of exposure to toxic environmental chemicals. Int J Gynecol Obstet. 2015; 131(3): 219-225. DOI: 10.1016/j.ijgo.2015.09.002.

2. Kahn LG., Trasande L. Environmental toxicant exposure and hypertensive disorders of pregnancy: Recent findings. Curr Hypertens Rep. 2018; 20: 87. DOI: 10.1007/s11906-018-0888-5.

3. Albouy-Llaty M, Limousi F, Carles C, Dupuis A, Rabouan S, Mignot V. Association between exposure to endocrine disruptors in drinking water and preterm birth, taking neighborhood deprivation into account: A historic cohort study. Int J Environ Res Public Health. 2016; 13(796): 2-16. DOI: 10.3390/ijerph13080796.

4. Grossman DS, Slusky DJG. The impact of the Flint water crisis on fertility. Demography. 2019; 56(6): 2005–2031. DOI: 10.1007/s13524-019-00831-0.

5. Abad M. Malekafzali CH. Simbar M. Mosaavi HS. Khoei EM. Association between electromagnetic field exposure and abortion in pregnant women living in Tehran. Iran J Reprod Biomed. 2016; 14(5): 347-354.

6. Doherty BT, Engel SM, Buckley JP, Silva MJ, Calafat AM, Wolff MS. Prenatal phthalate biomarker concentrations and performance on the Bayley Scale of Infant Development-II in a population of young urban children. Environ Res. 2017; 152: 51-58. DOI: 10.1016/j.envres.2016.09.021.

7. Vabre P, Gatimel N, Moreau J, et al. Environmental pollutants, a possible etiology for premature ovarian insufficiency: A narrative review of animal and human data. Environ Health. 2017; 16: 37. DOI: 10.1186/s12940-017-0242-4.

8. Botton J, Philippat C, Calafat AM, Carles S, Charles MA, Slama R, et al. Phthalate pregnancy exposure and male offspring growth from the intra-uterine period to five years of age. Environ Res. 2016; 151: 601-609. DOI: 10.1016/j.envres.2016.08.033.

9. Marsillach J, Costa G, Furlong CE. Paraoxonase-1 and early-life environmental exposures. Ann Glob Health. 2016; 82(1): 100-110. DOI: 10.1016/j.aogh.2016.01.009.

10. Chang S, Park S, Chung C. Effect of Taegyo-focused prenatal education on maternal-fetal attachment and self-efficacy related to childbirth. J Korean Acad Nurs. 2004; 34(8): 1409-1415. DOI: 10.4040/jkan.2004.34.8.1409.

11. Jeong GH, Kim HK. Pro-environmental health behaviour and educational needs among pregnant women: A cross-sectional survey. J Adv Nurs. 2020; 76(7): 1638-1646. DOI: 10.1111/jan.14346.

12. Rosas LG, Trujillo C, Camacho J, Madrigal D, Bradman A, Eskenazi B. Acceptability of health information technology aimed at environmental health education in a prenatal clinic. Patient Edu Couns. 2014; 97(2): 244-7. DOI: 10.1016/j.pec.2014.07.018.

13. Osanan GC, Vidarte MFE, Ludmir J. Do not forget our pregnant women during the COVID-19 pandemic. Women Health. 2020; 60(9): 959-962. DOI: 10.1080/03630242.2020.1789264.

14. Sinclair P, Kable A, Levett-Jones T. The effectiveness of internet-based e-learning on clinician behavior and patient outcomes: A systematic review protocol. JBI Database System Rev Implement Rep. 2015; 13(1): 52-64. DOI: 10.11124/jbisrir-2015-1919.

15. Abuidhail J, Mayan L, Jaradat D. Evaluating effects of prenatal web-based breastfeeding education for pregnant mothers in their third trimester of pregnancy: Prospective randomized control trial. Midwifery. 2019; 69: 143-149. DOI: 10.1016/j.midw.2018.11.015.

16. Chan KL, Chen M. Effects of social media and mobile health apps on pregnancy care: Meta-analysis. JMIR mHealth uHealth. 2019; 7(1): e11836. DOI: 10.2196/11836.
