# Supplementary material for: The effects of environmental prenatal program on environmental health perception and behavior using internet-based intervention in South Korea: A non-randomized controlled study
Source: PLoS One. 2022 Nov 15;17(11):e0277501. doi: 10.1371/journal.pone.0277501 (PMC9665389; doi:10.1371/journal.pone.0277501)
Supplement: Attachment — Submitted filename: PONE.docx [file pone.0277501.s005.docx]

PONE-D-22-04403

The effects of environmental prenatal program on environmental health perception and

behavior using internet-based intervention in Korea: a non-randomized controlled study

General Comments:

Prenatal counseling / educational programs rarely include education on how to protect pregnant populations and their unborn from environmental toxins. The goal of this research was to (1) develop an internet-based environmental prenatal program (IEPP) for pregnant women and (2) investigate the effect of an IEPP on environmental health perceptions, behaviors, and educational satisfaction. This is an interesting project and more research into how evidence-based prenatal programs reduce toxicant exposure during pregnancy is needed. However, there is some question concerning the homogeneity of the experimental and control groups.

ABSTRACT

The authors state: “Conclusion : The internet-based educational program can be the substitution for the face-to-face prenatal class to promote environmental health perceptions during pregnancy in the situation of the

pivotal prenatal program.”

INTRODUCTION

No suggestions

METHODS

Re: “The content of each of the four sessions included sections on 1) chemical hazards and micro-dust, 2)

125 environmental toxin and fetal health, 3) EDCs and electromagnetic waves, and 4) environmental hazards in the 126 postpartum period.” The pairing of content (e.g., chemical hazards with micro-dust and EDCs and electromagnetic) feels a bit counter intuitive.

The authors mention that “The overall CVI was .90 or higher, which was higher than the criterion

142 of .78 [21].” Is .78 a critical cutpoint. More elaboration on what is meant by this statement is needed

Re: “Fourth, a pilot intervention was conducted during the implementation stage before the main application of the 145 IEPP. In April 2021, an online lecture for 9 master's degree students was held for 2 hours through Zoom video 146 conferencing. The researchers discussed the relevance and effectiveness of the content.” So 9 master degree students were used to pilot the intervention. How is this a representative sample to pilot in compared to pregnant women targeted for recruitment during the intervention study?

Re: “Fifth, during the analysis stage, the intervention effects were evaluated in terms of environmental health

148 perceptions, behaviors, and educational satisfaction through a questionnaire survey.” Was this a pre-/post- survey or only post?

Section 3) Measurements

(1) Environmental health perceptions: primary outcomes pg 8 the first paragraph starting with “The severity scale includes 10 items across three subscales: 4 items on”chemicals” to the sentence starting as ”The barriers scale includes 10 items across 2 subscales” might be better captured in a table rather than text to aid the reader. This suggestion also applies to the (2) Environmental health behvarios: primary outcomes section.

Re: “4) Data collection 216 Data collection was requested by the heads of the health centers and the maternal and child health teams at 2 217 public health centers.” What do the authors mean by this? The study was directed by the public health centers? Further, the data collection section makes no mention of how participants were randomly allocated to each study arm.

Section 5) Research practice

Re: “(1) Step 1: Preliminary test 233 A pilot test was conducted to confirm the suitability of the questionnaire and to address problems with the 234 educational materials.” Can the authors provide more details on the pilot testing?

RESULTS

The control and experimental group appear to be different from each other, even if marginally not significant, based on age (experimental group slightly older), fewer children (experimental group slightly fewer), employment status (more experimental group employed compared to control). These differences may in part explain why the experimental group has higher risk perception of environmental toxins as a prenatal threat and therefore higher response efficacy in avoiding prenatal toxicant exposure. This is a limitation of the study.

DISCUSSION

This paragraph starting “Susceptibility refers to the degree to which a person believes” doesn’t serve to anchor the authors’ findings to the extant literature and reads more like an introductory paragraph. Consider revising to make specific to the study findings and existing literature.

This section might benefit more with the addition of a Future studies section that includes what another study that might motivate change in behavior + educational satisfaction might look like.
