# Supplementary material for: The effects of environmental prenatal program on environmental health perception and behavior using internet-based intervention in South Korea: A non-randomized controlled study
Source: PLoS One. 2022 Nov 15;17(11):e0277501. doi: 10.1371/journal.pone.0277501 (PMC9665389; doi:10.1371/journal.pone.0277501)
Supplement: Peer review history

# Author comment: Author response to previous submission

**DOI:** 10.1371/journal.pone.0277501.r001

29 Mar 2022

Attachment Submitted filename: 6_trendstatement_TREND_Checklist_LEPP2.pdf Click here for additional data file.

# Peer review: Decision Letter 0

**DOI:** 10.1371/journal.pone.0277501.r002

**Authors:** Clare Mc Fadden

14 Aug 2022

PONE-D-22-04403The effects of environmental prenatal program on environmental health perception and behavior using internet-based intervention in Korea: a non-randomized controlled studyPLOS ONE

Dear Dr. Jeong,

Thank you for submitting your manuscript to PLOS ONE. After careful consideration, we feel that it has merit but does not fully meet PLOS ONE’s publication criteria as it currently stands. Therefore, we invite you to submit a revised version of the manuscript that addresses the points raised during the review process. Please additional guidance from the editor below my signature. The manuscript has been evaluated by four reviewers, and their comments are available below.

The reviewers have raised a number of concerns that need attention. They request additional information on the pilot/preliminary study, the education content provided for the face-to-face program, and highlighted potential limitations of the study.  Could you please carefully revise the manuscript to address all comments raised?

Please submit your revised manuscript by Sep 28 2022 11:59PM. If you will need more time than this to complete your revisions, please reply to this message or contact the journal office at plosone@plos.org. When you're ready to submit your revision, log on to https://www.editorialmanager.com/pone/ and select the 'Submissions Needing Revision' folder to locate your manuscript file.

Please include the following items when submitting your revised manuscript:

- A rebuttal letter that responds to each point raised by the academic editor and reviewer(s). You should upload this letter as a separate file labeled 'Response to Reviewers'.
- A marked-up copy of your manuscript that highlights changes made to the original version. You should upload this as a separate file labeled 'Revised Manuscript with Track Changes'.
- An unmarked version of your revised paper without tracked changes. You should upload this as a separate file labeled 'Manuscript'.

If you would like to make changes to your financial disclosure, please include your updated statement in your cover letter. Guidelines for resubmitting your figure files are available below the reviewer comments at the end of this letter.

If applicable, we recommend that you deposit your laboratory protocols in protocols.io to enhance the reproducibility of your results. Protocols.io assigns your protocol its own identifier (DOI) so that it can be cited independently in the future. For instructions see: https://journals.plos.org/plosone/s/submission-guidelines#loc-laboratory-protocols. Additionally, PLOS ONE offers an option for publishing peer-reviewed Lab Protocol articles, which describe protocols hosted on protocols.io. Read more information on sharing protocols at https://plos.org/protocols?utm_medium=editorial-email&utm_source=authorletters&utm_campaign=protocols.

We look forward to receiving your revised manuscript.

Kind regards,

Clare Mc Fadden

Editorial Office

PLOS ONE

Journal Requirements:

When submitting your revision, we need you to address these additional requirements.

1. Please ensure that your manuscript meets PLOS ONE's style requirements, including those for file naming. The PLOS ONE style templates can be found at

https://journals.plos.org/plosone/s/file?id=wjVg/PLOSOne_formatting_sample_main_body.pdf and

https://journals.plos.org/plosone/s/file?id=ba62/PLOSOne_formatting_sample_title_authors_affiliations.pdf

2. Thank you for submitting your clinical trial to PLOS ONE and for providing the name of the registry and the registration number. The information in the registry entry suggests that your trial was registered after patient recruitment began. PLOS ONE strongly encourages authors to register all trials before recruiting the first participant in a study.

As per the journal’s editorial policy, please include in the Methods section of your paper:

1) your reasons for your delay in registering this study (after enrolment of participants started);

2) confirmation that all related trials are registered by stating: “The authors confirm that all ongoing and related trials for this drug/intervention are registered”.

3. Please provide additional details regarding participant consent. In the ethics statement in the Methods and online submission information, please ensure that you have specified what type you obtained (for instance, written or verbal, and if verbal, how it was documented and witnessed). If your study included minors, state whether you obtained consent from parents or guardians. If the need for consent was waived by the ethics committee, please include this information.

4. Thank you for stating in your Funding Statement:

“HKK received the fund.

This work was supported by the National Research Foundation of Korea (NRF) Grant funded by the Korea government (MIST) (No. 2020048449). URL: https://www.nrf.re.kr/eng/index.  The funders had no role in study design, data collection and analysis, decision to publish, or preparation of the manuscript.”

Please provide an amended statement that declares *all* the funding or sources of support (whether external or internal to your organization) received during this study, as detailed online in our guide for authors at http://journals.plos.org/plosone/s/submit-now.  Please also include the statement “There was no additional external funding received for this study.” in your updated Funding Statement.

Please include your amended Funding Statement within your cover letter. We will change the online submission form on your behalf.

5. Please include a caption for figure 1.

6. Please include captions for your Supporting Information files at the end of your manuscript, and update any in-text citations to match accordingly. Please see our Supporting Information guidelines for more information: http://journals.plos.org/plosone/s/supporting-information.

Additional Editor Comments (if provided):

1. Please disregard the request by Reviewer 1 to insert a PRISMA flow diagram

2. Table 1 provides details on the internet-based program. Please clarify if a similar program of content was provided for the face-to-face program. Please state if there are any discrepancies between the content of the internet-based and face-too-face programs.

3. Reviewer 3 noted that there are difference between the control and experimental groups that may be considered as a potential limitation of this study, despite not reaching statistical significance. Please ensure this is adequately discussed.

4. Please provide further information on the process of randomizing participants to each group, and please clarify if "participants were blinded to the intervention" and if so, how blinding was maintained given the circumstances of the intervention.

5. Please provide additional information on the preliminary/pilot testing of the questionnaire, as requested by Reviewer 3.

[Note: HTML markup is below. Please do not edit.]

Reviewers' comments:

Reviewer's Responses to Questions

**Comments to the Author**

1. Is the manuscript technically sound, and do the data support the conclusions?

The manuscript must describe a technically sound piece of scientific research with data that supports the conclusions. Experiments must have been conducted rigorously, with appropriate controls, replication, and sample sizes. The conclusions must be drawn appropriately based on the data presented. 

Reviewer #1: Yes

Reviewer #2: Partly

Reviewer #3: Partly

Reviewer #4: Yes

**********

2. Has the statistical analysis been performed appropriately and rigorously? 

Reviewer #1: I Don't Know

Reviewer #2: Yes

Reviewer #3: Yes

Reviewer #4: No

**********

3. Have the authors made all data underlying the findings in their manuscript fully available?

The PLOS Data policy requires authors to make all data underlying the findings described in their manuscript fully available without restriction, with rare exception (please refer to the Data Availability Statement in the manuscript PDF file). The data should be provided as part of the manuscript or its supporting information, or deposited to a public repository. For example, in addition to summary statistics, the data points behind means, medians and variance measures should be available. If there are restrictions on publicly sharing data—e.g. participant privacy or use of data from a third party—those must be specified.

Reviewer #1: Yes

Reviewer #2: Yes

Reviewer #3: No

Reviewer #4: Yes

**********

4. Is the manuscript presented in an intelligible fashion and written in standard English?

PLOS ONE does not copyedit accepted manuscripts, so the language in submitted articles must be clear, correct, and unambiguous. Any typographical or grammatical errors should be corrected at revision, so please note any specific errors here.

Reviewer #1: Yes

Reviewer #2: Yes

Reviewer #3: Yes

Reviewer #4: Yes

**********

5. Review Comments to the Author

Please use the space provided to explain your answers to the questions above. You may also include additional comments for the author, including concerns about dual publication, research ethics, or publication ethics. (Please upload your review as an attachment if it exceeds 20,000 characters)

Reviewer #1: There is a lot of interest on this study, particularly in pandemic situations. Internet-based interventions offer an interesting alternative to face-to-face interventions. There is a need to improve the process of designing, assessing and disseminating this type of intervention.

The option of ADDIE model is very interesting.

Speaking very generally, scientific litterature needs more publications on environmental health promotion and prenatal/perinatal educational programs.

I have just a couple of comments.

1- In order to better illustrate the literature review procedure, I propose to insert prisma flow diagram

2- Enrollment of pregnant women was not very clear. How could the participants possibly have been "blinded to the intervention" ? I assume that they know which group they are in (experimental or control group) due to the nature of the intervention. Could you provide further clarification on this process in the manuscript ?

3- By comparing the content of face-to-face intervention and internet-based environmental prenatal health program, we can clearly see that the latter focussed more on the environmental themes (Table 1). If educational content and objectives were similar in both programs, could you clarify it ?

4- Regarding the results, have you compared the post-test scores or the difference between post-test scores and pre-test scores ?

5- In discussion, you did not includ the choix of rPMT : good choice? alternatives ? ... (1 or 2 sentences with references will be sufficient)

6- In conclusion, maybe we are lacking perspectives to improve the internet-based intervention (through offring a smart gadget to reduce inequity, including fathers, ...)

7- It will get better if you could translate the data table.

Reviewer #2: The research study was about an important topic. The manuscript was well written. The theoretical framework should be thoroughly described in relation to the research study. The research hypotheses can be revised. The theoretical framework should also be described in relation to the findings of the study for the discussion section of the article. The limitations of the study should be addressed. Please also include robust sections about implications for practice and implications for future studies.

Reviewer #3: PONE-D-22-04403

The effects of environmental prenatal program on environmental health perception and

behavior using internet-based intervention in Korea: a non-randomized controlled study

General Comments:

Prenatal counseling / educational programs rarely include education on how to protect pregnant populations and their unborn from environmental toxins. The goal of this research was to (1) develop an internet-based environmental prenatal program (IEPP) for pregnant women and (2) investigate the effect of an IEPP on environmental health perceptions, behaviors, and educational satisfaction. This is an interesting project and more research into how evidence-based prenatal programs reduce toxicant exposure during pregnancy is needed. However, there is some question concerning the homogeneity of the experimental and control groups.

ABSTRACT

The authors state: “Conclusion : The internet-based educational program can be the substitution for the face-to-face prenatal class to promote environmental health perceptions during pregnancy in the situation of the

pivotal prenatal program.”

INTRODUCTION

No suggestions

METHODS

Re: “The content of each of the four sessions included sections on 1) chemical hazards and micro-dust, 2)

125 environmental toxin and fetal health, 3) EDCs and electromagnetic waves, and 4) environmental hazards in the 126 postpartum period.” The pairing of content (e.g., chemical hazards with micro-dust and EDCs and electromagnetic) feels a bit counter intuitive.

The authors mention that “The overall CVI was .90 or higher, which was higher than the criterion

142 of .78 [21].” Is .78 a critical cutpoint. More elaboration on what is meant by this statement is needed

Re: “Fourth, a pilot intervention was conducted during the implementation stage before the main application of the 145 IEPP. In April 2021, an online lecture for 9 master's degree students was held for 2 hours through Zoom video 146 conferencing. The researchers discussed the relevance and effectiveness of the content.” So 9 master degree students were used to pilot the intervention. How is this a representative sample to pilot in compared to pregnant women targeted for recruitment during the intervention study?

Re: “Fifth, during the analysis stage, the intervention effects were evaluated in terms of environmental health

148 perceptions, behaviors, and educational satisfaction through a questionnaire survey.” Was this a pre-/post- survey or only post?

Section 3) Measurements

(1) Environmental health perceptions: primary outcomes pg 8 the first paragraph starting with “The severity scale includes 10 items across three subscales: 4 items on”chemicals” to the sentence starting as ”The barriers scale includes 10 items across 2 subscales” might be better captured in a table rather than text to aid the reader. This suggestion also applies to the (2) Environmental health behvarios: primary outcomes section.

Re: “4) Data collection 216 Data collection was requested by the heads of the health centers and the maternal and child health teams at 2 217 public health centers.” What do the authors mean by this? The study was directed by the public health centers? Further, the data collection section makes no mention of how participants were randomly allocated to each study arm.

Section 5) Research practice

Re: “(1) Step 1: Preliminary test 233 A pilot test was conducted to confirm the suitability of the questionnaire and to address problems with the 234 educational materials.” Can the authors provide more details on the pilot testing?

RESULTS

The control and experimental group appear to be different from each other, even if marginally not significant, based on age (experimental group slightly older), fewer children (experimental group slightly fewer), employment status (more experimental group employed compared to control). These differences may in part explain why the experimental group has higher risk perception of environmental toxins as a prenatal threat and therefore higher response efficacy in avoiding prenatal toxicant exposure. This is a limitation of the study.

DISCUSSION

This paragraph starting “Susceptibility refers to the degree to which a person believes” doesn’t serve to anchor the authors’ findings to the extant literature and reads more like an introductory paragraph. Consider revising to make specific to the study findings and existing literature.

This section might benefit more with the addition of a Future studies section that includes what another study that might motivate change in behavior + educational satisfaction might look like.

Reviewer #4: Interesting article.

Line 163: phrase in future tense: instead of “participated” suggest “indicated that they were willing to participate”

Line 169: it is unclear what was planned and what was done. Were the more than 26 subjects recruited in each group as there were 33 and 34 subjects who began the study? This language needs clarification.

Perhaps the sentence at line 171-2 would be better in the results, leaving this section to be what was planned/ intended? Or greater clarity about what was intended and what was done, explaining the difference and giving a reason.

Line 177: leaving out subjects who did not complete the intervention may lead to a larger difference between groups if the intervention works than would be possible in a real world setting (if reasons for not completing the intervention were even partly related to the intervention). Was this considered? If so, please add to the text.

Line 221: advanced?

Line 304+: were these analyses adjusted for baseline levels? Would it have been better to analyse differences from baseline rather than post test results? How were baseline differences in the measures accounted for?

Discussion. The intervention is delivered after the first trimester in most cases, thus minimising environmental exposures that are potentially harm less at this stage of pregnancy will not be useful to this pregnancy. Is this an issue that could be improved upon?

**********

6. PLOS authors have the option to publish the peer review history of their article (what does this mean?). If published, this will include your full peer review and any attached files.

If you choose “no”, your identity will remain anonymous but your review may still be made public.

**Do you want your identity to be public for this peer review?** For information about this choice, including consent withdrawal, please see our Privacy Policy.

Reviewer #1: No

Reviewer #2: No

Reviewer #3: No

Reviewer #4: No

**********

[NOTE: If reviewer comments were submitted as an attachment file, they will be attached to this email and accessible via the submission site. Please log into your account, locate the manuscript record, and check for the action link "View Attachments". If this link does not appear, there are no attachment files.]

While revising your submission, please upload your figure files to the Preflight Analysis and Conversion Engine (PACE) digital diagnostic tool, https://pacev2.apexcovantage.com/. PACE helps ensure that figures meet PLOS requirements. To use PACE, you must first register as a user. Registration is free. Then, login and navigate to the UPLOAD tab, where you will find detailed instructions on how to use the tool. If you encounter any issues or have any questions when using PACE, please email PLOS at figures@plos.org. Please note that Supporting Information files do not need this step.

Attachment Submitted filename: PONE.docx Click here for additional data file.

# Author comment: Author response to Decision Letter 0

**DOI:** 10.1371/journal.pone.0277501.r003

12 Sep 2022

The effects of environmental prenatal program on environmental health perception and behavior using internet-based intervention in Korea: a non-randomized controlled study

Manuscript ID : PONE-D-22-04403

Journal Requirements Changes made to Article Page

1.Please ensure that your manuscript meets PLOS ONE's style requirements, including those for file naming. Thank you for your comments. We changed file names, heading, font size, and inserted supporting information and the caption of the figure according to PLOS ONE’s guidelines.

“(Fig 1)

Fig 1. Flow diagram of the research process. 

8

2.Thank you for submitting your clinical trial to PLOS ONE and for providing the name of the registry and the registration number. The information in the registry entry suggests that your trial was registered after patient recruitment began. PLOS ONE strongly encourages authors to register all trials before recruiting the first participant in a study

1) your reasons for your delay in registering this study (after enrolment of participants started) Registering clinical trials when the study begins to making information publicly available fulfills a number of purposes and benefits readers. However, COVID-19 regulation for quarantine made all the face-to-face prenatal education class to change abruptly in the step of planning this study. Instead of the registration clinical trials, researchers discussed and performed this project according to the protocol made by ourselves. 12

2) confirmation that all related trials are registered by stating: “The authors confirm that all ongoing and related trials for this drug/intervention are registered” We added this statement at the proper section.

“The authors confirm that all ongoing and related trials for this intervention are registered.” 12

3. Please provide additional details regarding participant consent. In the ethics statement in the Methods and online submission information, please ensure that you have specified what type you obtained (for instance, written or verbal, and if verbal, how it was documented and witnessed). If your study included minors, state whether you obtained consent from parents or guardians. If the need for consent was waived by the ethics committee, please include this information. “In order to protect the rights of the participant, informed consent documentations with participants’ signatures were obtained after the purpose and methods of the study, the benefits and drawbacks of participation in the study, privacy and confidentiality, and the possibility of refusal and withdrawal from the study before data collection were explained. There was no culturally minority or under 18 aged participants.” 11-12

This work was supported by the National Research Foundation of Korea (NRF) Grant funded by the Korea government (MIST) (No. 2020048449). URL: https://www.nrf.re.kr/eng/index. The funders had no role in study design, data collection and analysis, decision to publish, or preparation of the manuscript.”

Please provide an amended statement that declares *all* the funding or sources of support (whether external or internal to your organization) received during this study, as detailed online in our guide for authors at http://journals.plos.org/plosone/s/submit-now. Please also include the statement “There was no additional external funding received for this study.” in your updated Funding Statement.

Please include your amended Funding Statement within your cover letter. We will change the online submission form on your behalf. This study was approved by the Bioethics Committee of the Kongju National University(KNU-IRB-2021-04). This work was supported by the National Research Foundation of Korea (NRF) Grant funded by the Korea government (MIST) (No. 2020048449, URL: https://doi.org/10.13039/501100003725). The NRF had no role in study design, data collection and analysis, decision to publish, or preparation of the manuscript. There was no additional external or internal funding received for this study. Title page

Cover letter

5. Please include a caption for figure 1 Fig 1. Flow diagram of the research process. 8

6. Please include captions for your Supporting Information files at the end of your manuscript, and update any in-text citations to match accordingly. Please see our Supporting Information guidelines for more information: http://journals.plos.org/plosone/s/supporting-information. Supporting information

 S1 Dataset 1. Raw response data from participants.

 23

Additional Editor Comments Changes made to Article Page

1. Please disregard the request by Reviewer 1 to insert a PRISMA flow diagram Thank you for your comments. 

2. Table 1 provides details on the internet-based program. Please clarify if a similar program of content was provided for the face-to-face program. Please state if there are any discrepancies between the content of the internet-based and face-too-face programs. We mentioned the program for control group at page 11. However, we added the contents for control group in this part in the light of the discrepancies between two groups.

“For the control group, treatment as usual (TAU) was performed as knowledge of pregnancy, delivery, postpartum care, and neonatal care during four weeks through face-to-face education in small group under ten participants at the birth class in the public health center” 

5

3. Reviewer 3 noted that there are difference between the control and experimental groups that may be considered as a potential limitation of this study, despite not reaching statistical significance. Please ensure this is adequately discussed. We analyze the potential differences from age, number of children, and employment status using ANCOVA adjusted these three variables. The results and discussion parts were changed in this light.

“After intervention of the program, environmental severity (F=17.96, p<.001), response efficacy (F=15.69, p<.001), and total environmental perception (F=7.80, p=.008) were higher in the experimental group than in the control group.”

“The Shapiro-Wilk test was used to test the normality of variables, and the ANCOVA (Analysis of Covariance) and t-test were performed to test the difference in posttest variables between the control and experimental groups.”

“The F score was derived from an ANCOVA with age, number of children, and dummy variables of employment status.”

“(1) Primary outcome effects of the IEPP on environmental health perceptions

Hypothesis 1 was partially supported. The severity score was significantly higher in the IEPP group than in the control group (40.92±3.93 and 45.76±3.47) (F=17.96, p<.001). The response efficacy score was significantly higher in the IEPP group than in the control group (40.25±5.40 and 45.88±4.42) (F=15.69, p<.001). In addition, the total score was significantly in the IEPP group than in the control group (236.83±22.80 and 250.68±15.43) (F=7.80, p=.008). There were no statistically significant differences with regard to susceptibility (43.92±4.74 and 45.92±4.46) (F=2.09, p=.155), self-efficacy (45.67±8.76 and 47.80±6.72) (F=0.62, p=.435), benefits (30.04±4.81 and 32.92±4.04) (F=2.82, p=.054), and barriers (32.21±4.32 and 32.40±4.88) (F=0.11, p=.741) between the control and experimental groups (Table 3).

(2) Primary outcome effects of the IEPP on environmental health behaviors

Hypothesis 2 was rejected. There were no statistically significant differences in personal health behaviors (58.79±7.80 and 61.32±8.29) (F=0.34, p=.562), community health behaviors (58.25±9.26 and 57.00±7.50) (F=0.91, p=.344), or the total score (119.67±13.56 and 118.32±14.10) (F=0.05, p=.815) between the control and experimental groups (Table 3). 

2

11

14

Table 3.

13-14

4. Please provide further information on the process of randomizing participants to each group, and please clarify if "participants were blinded to the intervention" and if so, how blinding was maintained given the circumstances of the intervention. “The unit of assignment was individuals, and all participants in the class were included to minimize the potential bias resulting from non-randomization. The enrollment and intervention were independently performed apart region and the participants did not know whether they were included experimental or control group. The participants were single blinded to the intervention even when researchers were not blinded to the assignment conditions.” 8

5. Please provide additional information on the preliminary/pilot testing of the questionnaire, as requested by Reviewer 3. The preliminary test was performed to pregnant women. The terms were changed pilot to exercise of intervention.

“Fourth, a rehearsal of intervention for consultation was conducted during the implementation stage before the main application of the IEPP.”

“The program was conducted at a public health center in G City, and educational materials were presented to six pregnant women aged from 22 to 30 at a prenatal class in April 2021.” 

7

10

Reviewers' comments #1 Changes made to Article Page

1.There is a lot of interest on this study, particularly in pandemic situations. Internet-based interventions offer an interesting alternative to face-to-face interventions. There is a need to improve the process of designing, assessing and disseminating this type of intervention.

The option of ADDIE model is very interesting. Thank you for your invaluable comments. 

2.Enrollment of pregnant women was not very clear. How could the participants possibly have been "blinded to the intervention" ? I assume that they know which group they are in (experimental or control group) due to the nature of the intervention. Could you provide further clarification on this process in the manuscript ? “The unit of assignment was individuals, and all participants in the class were included to minimize the potential bias resulting from non-randomization. The enrollment and intervention were independently performed apart region and the participants did not know whether they were included experimental or control group. The participants were single blinded to the intervention even when researchers were not blinded to the assignment conditions.” 8

3.By comparing the content of face-to-face intervention and internet-based environmental prenatal health program, we can clearly see that the latter focussed more on the environmental themes (Table 1). If educational content and objectives were similar in both programs, could you clarify it ? “For the control group, treatment as usual (TAU) was performed as knowledge of pregnancy, delivery, postpartum care, and neonatal care during four weeks through face-to-face education in small group under ten participants at the birth class in the public health center.” 5

4.Regarding the results, have you compared the post-test scores or the difference between post-test scores and pre-test scores ? We compared the post-test score between experimental and control group.

“The Shapiro-Wilk test was used to test the normality of variables, and the ANCOVA (Analysis of Covariance) and t-test were performed to test the difference in posttest variables between the control and experimental groups.” 

11

5.In discussion, you did not includ the choix of rPMT : good choice? alternatives ? ... (1 or 2 sentences with references will be sufficient) “Rogers [20] explained health behaviors were motivated through perception of the benefit and barriers in the internal process of severity, susceptibility, response efficacy, and self-efficacy in advance [20]. The results of this study showed the environmental behaviors were not fully motivated but participants adopted the emotional factors and behavioral appraisal related environmental threat.” 15

6.In conclusion, maybe we are lacking perspectives to improve the internet-based intervention (through offring a smart gadget to reduce inequity, including fathers, ...) “We suggest that offering smart gadgets to vulnerable population to provide equal opportunities in the pandemic era. The internet-based education can be extended to family participatory program with husband of pregnant women because of merits space and time.” 18

7.It will get better if you could translate the data table. We translate and provide dataset in English version. Data file

Reviewers' comments #2 Changes made to Article Page

The research study was about an important topic. The manuscript was well written. The theoretical framework should be thoroughly described in relation to the research study. The research hypotheses can be revised. The theoretical framework should also be described in relation to the findings of the study for the discussion section of the article. 

The limitations of the study should be addressed. Please also include robust sections about implications for practice and implications for future studies. Thank you for your invaluable comments. We revised the manuscript through in the light of your suggestions.

“Rogers [20] explained health behaviors were motivated through perception of the benefit and barriers in the internal process of severity, susceptibility, response efficacy, and self-efficacy in advance [20]. The results of this study showed the environmental behaviors were not fully motivated but participants adopted the emotional factors and behavioral appraisal related environmental threat.”

“We suggest that offering smart gadgets to vulnerable population to provide equal opportunities in the pandemic era. The internet-based education can be extended to family participatory program with husband of pregnant women because of merits space and time.”

“Future studies are needed to verify the effectiveness of internet-based interventions and diversify the types of educational interventions, including online media, mobile applications, counseling, and mentoring, and regional expansion should be promoted to include a larger proportion of pregnant women.” 

15

18

18

Reviewers' comments #3 Changes made to Article Page

Prenatal counseling / educational programs rarely include education on how to protect pregnant populations and their unborn from environmental toxins. The goal of this research was to (1) develop an internet-based environmental prenatal program (IEPP) for pregnant women and (2) investigate the effect of an IEPP on environmental health perceptions, behaviors, and educational satisfaction. This is an interesting project and more research into how evidence-based prenatal programs reduce toxicant exposure during pregnancy is needed. However, there is some question concerning the homogeneity of the experimental and control groups. Thank you for your invaluable comments. We tried to analyze data in the focus of potential homogeneity problems. The ANOVA was used to control the age and employment status. 14

Table 3

ABSTRACT

The authors state: “Conclusion : The internet-based educational program can be the substitution for the face-to-face prenatal class to promote environmental health perceptions during pregnancy in the situation of the pivotal prenatal program.” “The internet-based educational program can be the alternative for the face-to-face prenatal class to promote environmental health perceptions during pregnancy in the pandemic situations”. 2

METHODS

Re: “The content of each of the four sessions included sections on 1) chemical hazards and micro-dust, 2) 

125 environmental toxin and fetal health, 3) EDCs and electromagnetic waves, and 4) environmental hazards in the 

126 postpartum period.” The pairing of content (e.g., chemical hazards with micro-dust and EDCs and electromagnetic) feels a bit counter intuitive. “The content of each of the four sessions included sections on 1) understanding chemical hazards and micro-dust, 2) fetal health problems induced environmental toxin, 3) protection behaviors from EDCs and electromagnetic waves during pregnancy, and 4) environmental health behaviors during postpartum.” 5

The authors mention that “The overall CVI was .90 or higher, which was higher than the criterion

142 of .78 [21].” Is .78 a critical cutpoint. More elaboration on what is meant by this statement is needed. “The item-level content validity index (I-CVI) were 1.00 which were higher than the criterion .78 [21]. The scale-level content validity index of usual agreement [S-CVI (ua)] was .90 and the scale-level content validity index averaging [S-CVI (ave)] was 1.00 which were higher than the criterion of .90 [21].” 6-7

Re: “Fourth, a pilot intervention was conducted during the implementation stage before the main application of the 

145 IEPP. In April 2021, an online lecture for 9 master's degree students was held for 2 hours through Zoom video 

146 conferencing. The researchers discussed the relevance and effectiveness of the content.” So 9 master degree students were used to pilot the intervention. How is this a representative sample to pilot in compared to pregnant women targeted for recruitment during the intervention study? The preliminary test was performed to pregnant women. The terms were changed pilot to exercise of intervention. The education to the master’s degree students was not pilot test but rehearsal and consultation.

“Fourth, a rehearsal of intervention for consultation was conducted during the implementation stage before the main application of the IEPP.”

“The program was conducted at a public health center in G City, and educational materials were presented to six pregnant women aged from 22 to 30 at a prenatal class in April 2021.” 

7

10

Re: “Fifth, during the analysis stage, the intervention effects were evaluated in terms of environmental health 

148 perceptions, behaviors, and educational satisfaction through a questionnaire survey.” Was this a pre-/post- survey or only post? Satisfaction can be asked at the end of the program because the satisfaction was regarding the education. Hence, we surveyed educational satisfaction only after intervention.

“We surveyed educational satisfaction in the post-test because .the satisfaction was regarding the intervention.” 

7

Section 3) Measurements

(1) Environmental health perceptions: primary outcomes pg 8 the first paragraph starting with “The severity scale includes 10 items across three subscales: 4 items on”chemicals” to the sentence starting as ”The barriers scale includes 10 items across 2 subscales” might be better captured in a table rather than text to aid the reader. This suggestion also applies to the (2) Environmental health behvarios: primary outcomes section. The total numbers of subscales were 22 and scales 10 which were complicate to present to the table. We discussed the presentation on the table and decide to explain in the measurement section. Table 3

Re: “4) Data collection 216 Data collection was requested by the heads of the health centers and the maternal and child health teams at 2 217 public health centers.” What do the authors mean by this? The study was directed by the public health centers? Further, the data collection section makes no mention of how participants were randomly allocated to each study arm. The role of health center head permitted recruiting, intervention, and survey.

“The experimental group was recruited at C city and all applicants met criterions assigned to attend an online antenatal class for a total of four sessions, and in April, July, September, and November 2021, four 2-hour education sessions were conducted once per week via Zoom video conferencing. The experimental group participated in a KakaoTalk group chat room for advance communication. The control group was recruited at G city and all applicants met criterions were assigned to attend a face-to-face prenatal class for a total of four sessions, and in May, July, September, and November 2021, four 2-hour training sessions were conducted in the education room at the public health center.” 10

Section 5) Research practice

Re: “(1) Step 1: Preliminary test 233 A pilot test was conducted to confirm the suitability of the questionnaire and to address problems with the 234 educational materials.” Can the authors provide more details on the pilot testing? “The program was conducted at a public health center in G City, and educational materials were presented to six pregnant women aged from 22 to 30 at a prenatal class in April 2021.” 10

RESULTS

The control and experimental group appear to be different from each other, even if marginally not significant, based on age (experimental group slightly older), fewer children (experimental group slightly fewer), employment status (more experimental group employed compared to control). These differences may in part explain why the experimental group has higher risk perception of environmental toxins as a prenatal threat and therefore higher response efficacy in avoiding prenatal toxicant exposure. This is a limitation of the study. We analyze the potential differences from age, number of children, and employment status using ANCOVA adjusted these three variables. The results and discussion parts were changed in this light.

 “After intervention of the program, environmental severity (F=17.96, p<.001), response efficacy (F=15.69, p<.001), and total environmental perception (F=7.80, p=.008) were higher in the experimental group than in the control group.”

“The Shapiro-Wilk test was used to test the normality of variables, and the ANCOVA (Analysis of Covariance) and t-test were performed to test the difference in posttest variables between the control and experimental groups.”

“The F score was derived from an ANCOVA with age, number of children, and dummy variables of employment status.”

“(1) Primary outcome effects of the IEPP on environmental health perceptions

Hypothesis 1 was partially supported. The severity score was significantly higher in the IEPP group than in the control group (40.92±3.93 and 45.76±3.47) (F=17.96, p<.001). The response efficacy score was significantly higher in the IEPP group than in the control group (40.25±5.40 and 45.88±4.42) (F=15.69, p<.001). In addition, the total score was significantly in the IEPP group than in the control group (236.83±22.80 and 250.68±15.43) (F=7.80, p=.008). There were no statistically significant differences with regard to susceptibility (43.92±4.74 and 45.92±4.46) (F=2.09, p=.155), self-efficacy (45.67±8.76 and 47.80±6.72) (F=0.62, p=.435), benefits (30.04±4.81 and 32.92±4.04) (F=2.82, p=.054), and barriers (32.21±4.32 and 32.40±4.88) (F=0.11, p=.741) between the control and experimental groups (Table 3).

The study limitation was also described regarding intervention adaptation period.

“The gestational age of participants was varied from first to third trimester even though the environmental toxin was more harmful in the early gestation because of the vulnerability of the embryo. We suggest the environmental intervention adopted in the first trimester pregnant women.” 

2

11

14

Table 3

13

18

DISCUSSION

This paragraph starting “Susceptibility refers to the degree to which a person believes” doesn’t serve to anchor the authors’ findings to the extant literature and reads more like an introductory paragraph. Consider revising to make specific to the study findings and existing literature.

This section might benefit more with the addition of a Future studies section that includes what another study that might motivate change in behavior + educational satisfaction might look like. “Susceptibility similar with vulnerability refers to participant’s perception of the likelihood that health problems will affect herself. In the health beliefs model, participants interpret susceptibility as mental paths, maximizing behavioral outcomes in a way that corresponds to their values and expectations [20].” 

“If the attractiveness of the reward is greater than the sum of severity and vulnerability, environmental behavior is more likely to be selected. Otherwise, compensation and benefits were not effective to motivate found environmental behaviors [30], 16

16

Reviewers' comments #4 Changes made to Article Page

Line 163: phrase in future tense: instead of “participated” suggest “indicated that they were willing to participate” Thank you for your invaluable comments. We revised the manuscript through in the light of your suggestions.

“3) women who indicated that they were willing to participate for the full 4 weeks”, 

7

Line 169: it is unclear what was planned and what was done. Were the more than 26 subjects recruited in each group as there were 33 and 34 subjects who began the study? This language needs clarification.

Perhaps the sentence at line 171-2 would be better in the results, leaving this section to be what was planned/ intended? Or greater clarity about what was intended and what was done, explaining the difference and giving a reason. We clarified these sentences to method section(participants). 

“In the pre-test, the experimental group started including 34 people enrolled in the study, and the control group including 33 people. In the posttest, final participants were included 25 out of 34 subjects from the experimental group and 24 out of 33 subjects from the control group, totaling 49 participants.” 

8

Line 177: leaving out subjects who did not complete the intervention may lead to a larger difference between groups if the intervention works than would be possible in a real world setting (if reasons for not completing the intervention were even partly related to the intervention). Was this considered? If so, please add to the text. “The drop-out rates and reasons were similar in the both group. The drop out reasons were personal reasons, household chores, job, obstetric regular check, and internet access problem in the experimental group and personal reasons, household chores, and incomplete post-test survey in the control group (Fig 1).” 8

Line 221: advanced? “for question and answer communication” 10

Line 304+: were these analyses adjusted for baseline levels? Would it have been better to analyse differences from baseline rather than post test results? How were baseline differences in the measures accounted for? We analyze the potential differences from age, number of children, and employment status using ANCOVA adjusted these three variables. The results and discussion parts were changed in this light.

 “After intervention of the program, environmental severity (F=17.96, p<.001), response efficacy (F=15.69, p<.001), and total environmental perception (F=7.80, p=.008) were higher in the experimental group than in the control group.”

“The Shapiro-Wilk test was used to test the normality of variables, and the ANCOVA (Analysis of Covariance) and t-test were performed to test the difference in posttest variables between the control and experimental groups.”

“The F score was derived from an ANCOVA with age, number of children, and dummy variables of employment status.”

“(1) Primary outcome effects of the IEPP on environmental health perceptions

Hypothesis 1 was partially supported. The severity score was significantly higher in the IEPP group than in the control group (40.92±3.93 and 45.76±3.47) (F=17.96, p<.001). The response efficacy score was significantly higher in the IEPP group than in the control group (40.25±5.40 and 45.88±4.42) (F=15.69, p<.001). In addition, the total score was significantly in the IEPP group than in the control group (236.83±22.80 and 250.68±15.43) (F=7.80, p=.008). There were no statistically significant differences with regard to susceptibility (43.92±4.74 and 45.92±4.46) (F=2.09, p=.155), self-efficacy (45.67±8.76 and 47.80±6.72) (F=0.62, p=.435), benefits (30.04±4.81 and 32.92±4.04) (F=2.82, p=.054), and barriers (32.21±4.32 and 32.40±4.88) (F=0.11, p=.741) between the control and experimental groups (Table 3).

2

11

14

Table 3

13

Discussion. The intervention is delivered after the first trimester in most cases, thus minimising environmental exposures that are potentially harm less at this stage of pregnancy will not be useful to this pregnancy. Is this an issue that could be improved upon? The study limitation was also described regarding intervention adaptation period.

“The gestational age of participants was varied from first to third trimester even though the environmental toxin was more harmful in the early gestation because of the vulnerability of the embryo. We suggest the environmental intervention adopted in the first trimester pregnant women.” 

18

Attachment Submitted filename: Author Response to Reviewers_LEPP_Sep09.docx Click here for additional data file.

# Peer review: Decision Letter 1

**DOI:** 10.1371/journal.pone.0277501.r004

**Authors:** George Vousden

31 Oct 2022

The effects of environmental prenatal program on environmental health perception and behavior using internet-based intervention in South Korea: a non-randomized controlled study

PONE-D-22-04403R1

Dear Dr. Jeong,

We’re pleased to inform you that your manuscript has been judged scientifically suitable for publication and will be formally accepted for publication once it meets all outstanding technical requirements.

Within one week, you’ll receive an e-mail detailing the required amendments. When these have been addressed, you’ll receive a formal acceptance letter and your manuscript will be scheduled for publication.

An invoice for payment will follow shortly after the formal acceptance. To ensure an efficient process, please log into Editorial Manager at http://www.editorialmanager.com/pone/, click the 'Update My Information' link at the top of the page, and double check that your user information is up-to-date. If you have any billing related questions, please contact our Author Billing department directly at authorbilling@plos.org.

If your institution or institutions have a press office, please notify them about your upcoming paper to help maximize its impact. If they’ll be preparing press materials, please inform our press team as soon as possible -- no later than 48 hours after receiving the formal acceptance. Your manuscript will remain under strict press embargo until 2 pm Eastern Time on the date of publication. For more information, please contact onepress@plos.org.

Kind regards,

George Vousden

Staff Editor

PLOS ONE

Additional Editor Comments (optional):

Reviewers' comments:

Reviewer's Responses to Questions

**Comments to the Author**

1. If the authors have adequately addressed your comments raised in a previous round of review and you feel that this manuscript is now acceptable for publication, you may indicate that here to bypass the “Comments to the Author” section, enter your conflict of interest statement in the “Confidential to Editor” section, and submit your "Accept" recommendation.

Reviewer #1: All comments have been addressed

Reviewer #2: (No Response)

Reviewer #4: (No Response)

**********

2. Is the manuscript technically sound, and do the data support the conclusions?

The manuscript must describe a technically sound piece of scientific research with data that supports the conclusions. Experiments must have been conducted rigorously, with appropriate controls, replication, and sample sizes. The conclusions must be drawn appropriately based on the data presented. 

Reviewer #1: Yes

Reviewer #2: Yes

Reviewer #4: Yes

**********

3. Has the statistical analysis been performed appropriately and rigorously? 

Reviewer #1: (No Response)

Reviewer #2: Yes

Reviewer #4: Yes

**********

4. Have the authors made all data underlying the findings in their manuscript fully available?

The PLOS Data policy requires authors to make all data underlying the findings described in their manuscript fully available without restriction, with rare exception (please refer to the Data Availability Statement in the manuscript PDF file). The data should be provided as part of the manuscript or its supporting information, or deposited to a public repository. For example, in addition to summary statistics, the data points behind means, medians and variance measures should be available. If there are restrictions on publicly sharing data—e.g. participant privacy or use of data from a third party—those must be specified.

Reviewer #1: Yes

Reviewer #2: Yes

Reviewer #4: (No Response)

**********

5. Is the manuscript presented in an intelligible fashion and written in standard English?

PLOS ONE does not copyedit accepted manuscripts, so the language in submitted articles must be clear, correct, and unambiguous. Any typographical or grammatical errors should be corrected at revision, so please note any specific errors here.

Reviewer #1: (No Response)

Reviewer #2: Yes

Reviewer #4: (No Response)

**********

6. Review Comments to the Author

Please use the space provided to explain your answers to the questions above. You may also include additional comments for the author, including concerns about dual publication, research ethics, or publication ethics. (Please upload your review as an attachment if it exceeds 20,000 characters)

Reviewer #1: (No Response)

Reviewer #2: I enjoyed reading your manuscript. You have made good improvements. The theoretical framework can be discussed in greater depth. I think the conclusion/discussion section can be expanded and can include more information about health care implications and implications for future studies.

Reviewer #4: Thank you for the alterations, I enjoyed reading this.

Line 173 on track changes: use "included".

**********

7. PLOS authors have the option to publish the peer review history of their article (what does this mean?). If published, this will include your full peer review and any attached files.

If you choose “no”, your identity will remain anonymous but your review may still be made public.

**Do you want your identity to be public for this peer review?** For information about this choice, including consent withdrawal, please see our Privacy Policy.

Reviewer #1: No

Reviewer #2: No

Reviewer #4: No

**********

# Editor report: Acceptance letter

**DOI:** 10.1371/journal.pone.0277501.r005

**Authors:** George Vousden

4 Nov 2022

PONE-D-22-04403R1 

The effects of environmental prenatal program on environmental health perception and behavior using internet-based intervention in South Korea: a non-randomized controlled study 

Dear Dr. Jeong:

I'm pleased to inform you that your manuscript has been deemed suitable for publication in PLOS ONE. Congratulations! Your manuscript is now with our production department. 

If your institution or institutions have a press office, please let them know about your upcoming paper now to help maximize its impact. If they'll be preparing press materials, please inform our press team within the next 48 hours. Your manuscript will remain under strict press embargo until 2 pm Eastern Time on the date of publication. For more information please contact onepress@plos.org.

If we can help with anything else, please email us at plosone@plos.org. 

Thank you for submitting your work to PLOS ONE and supporting open access. 

Kind regards, 

PLOS ONE Editorial Office Staff

on behalf of

Dr. George Vousden 

Staff Editor

PLOS ONE
